# Exploring links between 2‐oxoglutarate‐dependent oxygenases and Alzheimer's disease

**DOI:** 10.1002/alz.12733

**Published:** 2022-07-19

**Authors:** Haotian Liu, Yong Xie, Xia Wang, Martine I. Abboud, Chao Ma, Wei Ge, Christopher J. Schofield

**Affiliations:** ^1^ State Key Laboratory of Medical Molecular Biology & Department of Immunology Institute of Basic Medical Sciences Chinese Academy of Medical Sciences School of Basic Medicine Peking Union Medical College Beijing China; ^2^ National Clinical Research Center for Orthopedics Sports Medicine & Rehabilitation Department of Orthopedics General Hospital of Chinese PLA Beijing China; ^3^ The Chemistry Research Laboratory Department of Chemistry and the Ineos Oxford Institute for Antimicrobial Research University of Oxford Oxford UK; ^4^ Department of Human Anatomy, Histology and Embryology Neuroscience Center National Human Brain Bank for Development and Function Institute of Basic Medical Sciences Chinese Academy of Medical Sciences, School of Basic Medicine Peking Union Medical College Beijing China

**Keywords:** Alzheimer's disease, epigenetics, hydroxylases, hypoxia‐inducible factor, JmjC demethylases, synapse loss, 2‐oxoglutarate/alpha‐ketoglutarate dependent oxygenases

## Abstract

Hypoxia, that is, an inadequate oxygen supply, is linked to neurodegeneration and patients with cardiovascular disease are prone to Alzheimer's disease (AD). 2‐Oxoglutarate and ferrous iron‐dependent oxygenases (2OGDD) play a key role in the regulation of oxygen homeostasis by acting as hypoxia sensors. 2OGDD also have roles in collagen biosynthesis, lipid metabolism, nucleic acid repair, and the regulation of transcription and translation. Many biological processes in which the >60 human 2OGDD are involved are altered in AD patient brains, raising the question as to whether 2OGDD are involved in the transition from normal aging to AD. Here we give an overview of human 2OGDD and critically discuss their potential roles in AD, highlighting possible relationships with synapse dysfunction/loss. 2OGDD may regulate neuronal/glial differentiation through enzyme activity‐dependent mechanisms and modulation of their activity has potential to protect against synapse loss. Work linking 2OGDD and AD is at an early stage, especially from a therapeutic perspective; we suggest integrated pathology and in vitro discovery research to explore their roles in AD is merited. We hope to help enable long‐term research on the roles of 2OGDD and, more generally, oxygen/hypoxia in AD. We also suggest shorter term empirically guided clinical studies concerning the exploration of 2OGDD/oxygen modulators to help maintain synaptic viability are of interest for AD treatment.

## 1 INTRODUCTIVE NARRATIVE

### 1.1 Background

Alzheimer's disease (AD) is the most common cause of dementia, accounting for an estimated 60% to 80% of cases of dementia.^1^ In 2019, Alzheimer's Disease International estimated that >50 million people suffer from dementia globally, a figure set to increase to 152 million by 2050.^2^ A human develops dementia every three seconds and the current annual cost of dementia is estimated at US $1 trillion, a figure set to double by 2030.^2^ AD thus imposes severe physical and psychological burdens on patients and their families and places immense financial strains on health‐care systems and societies. The current SARS‐COV‐2 pandemic has highlighted this burden, with 8% of the total COVID‐19–related deaths being estimated to be of patients with AD.^3^

Despite decades of intensive research and some promising lines of investigation, effective treatments for dementia, including AD, are not yet available. The precise reasons for this are unclear but may reflect the many forms of dementia, lack of clear knowledge of factors affecting its progression, and knowledge that deterioration of brain function begins long before clinical diagnosis of dementia. The molecular basis of the neurobiology of dementia is not well understood. There is, however, evidence that massive loss of synapses in specific neural networks correlates with cognitive defects in dementia.^4^ However, the exact mechanisms underlying impairment of synapses/synaptic connectivity in dementia are poorly defined and none of the current pharmacological treatments addressing these are effective AD treatments.

### 1.2 2‐Oxoglutarate‐dependent oxygenases and AD: Conclusions and implications

Hypoxia, that is, the inadequate supply of atmospheric oxygen (O_2_, dioxygen) to organs, tissues, and cells, is linked to neurodegeneration,^5^ in particular in the context of synapse‐related factors converting normal aging into AD.^6^ Key roles for an ancient family of therapeutically tractable oxygen‐using enzymes, that is, the Fe(II) and 2‐oxoglutarate (2OG)‐dependent oxygenases (2OGDD), in the hypoxic response have been revealed.^7^ 2OGDD were first identified in studies on the biosynthesis of collagen, wherein they catalyze post‐translational hydroxylations of proline and lysine residues in procollagen.^8^ 2OG‐dependent prolyl‐hydroxylases (PHDs, prolyl hydroxylase domain enzymes) play key roles in regulating the hypoxic response by negatively regulating the hypoxia‐inducible transcription factors (HIFs) that mediate cellular and physiological responses working to ameliorate the effects of hypoxia. Importantly, there is evidence that PHD catalysis is limited by oxygen availability—in effect they act as sensors for hypoxia.^9^ A second type of 2OGDD, factor inhibiting HIF (FIH) also negatively regulates HIF, by catalyzing asparginyl‐hydroxylation, a modification that hinders the interaction of HIF with transcriptional coactivators.^9^, ^10^, ^11^

1 **RESEARCH IN CONTEXT**

1. **Systematic Review**: The authors reviewed the literature on human Fe (II) and 2‐oxoglutarate (2OG)‐dependent oxygenases (2OGDD) and their functions focusing on (potential) links to Alzheimer's disease (AD), especially those that are synapse related. Future research directions and methods that can be applied to test the roles of 2OGDD in AD and their suitability as drug targets are proposed. Several 2OGDD that have shown potential to treat AD are highlighted.
2. **Interpretation**: Our findings show the potential role of 2OGDD in synapse function, though research making direct causal links between 2OGDD malfunction and AD is presently lacking.
3. **Future Directions**: We believe long term integrated pathology and in vitro discovery research on the roles of 2OGDD and shorter term empirically guided clinical studies concerning 2OGDD inhibitors and other brain oxygen availability modulators in AD treatment are necessary.

**FIGURE 1:**
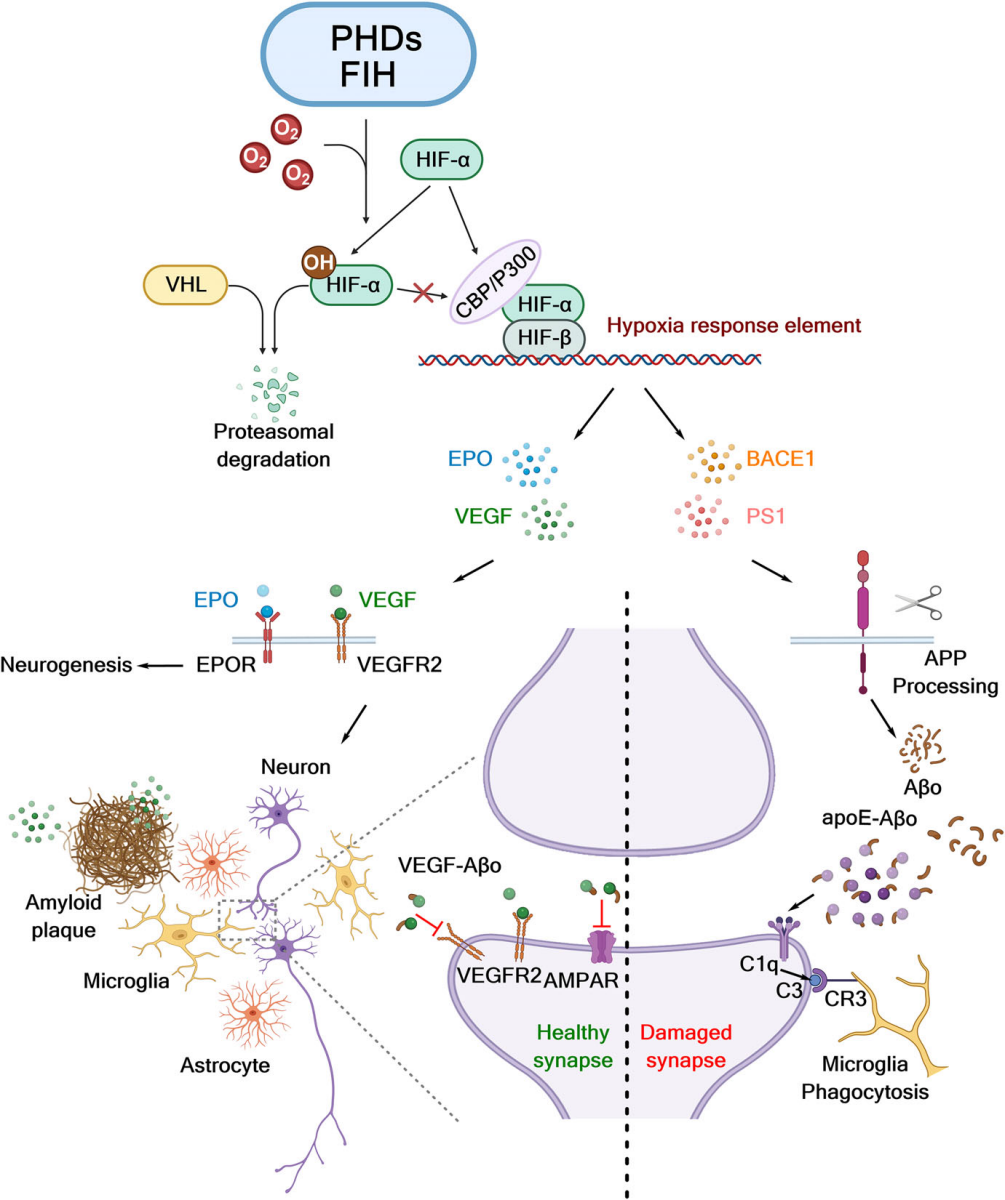
Potential links between HIFs, PHDs, FIH and the synapse. Levels of HIF‐α, but not HIF‐β, are regulated by the PHDs. In humans there are three PHDs (1–3) and three HIF‐α isoforms. Evidence from PHD‐deficient mouse models, cellular studies, and clinical work support the roles of the PHDs in regulating levels of EPO and VEGF (and potentially other genes). EPO and VEGF are HIF target genes and are neuroprotective factors, of relevance to AD. EPO could drive neuroplasticity and neurogenesis via EPO/EPOR signaling. VEGF accumulates in Aβ plaques and rescues Aβo‐induced impairment in synapse through directly interacting with Aβo. HIF‐1α is reported to increase APP processing and Aβ generation by promoting expression of β‐ and γ‐secretases in the amyloidogenic pathway and inhibiting α‐secretases in the non‐amyloidogenic pathway. Aβ monomers then assemble to form a variety of soluble Aβ oligomers, which have been implicated in the synaptic loss observed in AD patients. The roles of HIF in AD are therefore likely complex. (The figure was created with biorender.com.) Aβ, amyloid beta; AD, Alzheimer's disease; APP, amyloid precursor protein; EPO, erythropoietin; FIH, factor‐inhibiting hypoxia‐inducible transcription factors; HIF, hypoxia‐inducible transcription factors; PHD, prolyl‐hydroxylases; VEGF, vascular endothelial growth factor

HIF target genes include vascular endothelial growth factor (*VEGF*) and erythropoietin (*EPO*), which are neuroprotective and have roles in neurogenesis and synapse protection.^12^, ^13^ Importantly, the PHDs have been shown to be amenable to small‐molecule inhibition. It should be noted that the clinically used PHD inhibitors for treatment of anemia in chronic kidney disease appear to be safe, as far as is known, despite the perceived pleiotropic nature of HIF target genes (though, e.g., excessive EPO upregulation is, of course, toxic). We formulate a verifiable hypothesis that PHD/FIH mediated regulation of the hypoxia response can, on the one hand, promote the expression of proteases involved in processing amyloid precursor protein (APP; beta‐secretase 1 [BACE1] and presenilin 1 [PS1]), and hence, amyloid beta (Aβ) formation, resulting in microglia‐mediated synapse loss. On the other hand, the expression of several neuroprotective factors (VEGF and EPO) is upregulated by hypoxia/PHD/FIH inhibition, protecting synapses by competitively binding soluble Aβ oligomer (Aβo; Figure 1).

**FIGURE 2:**
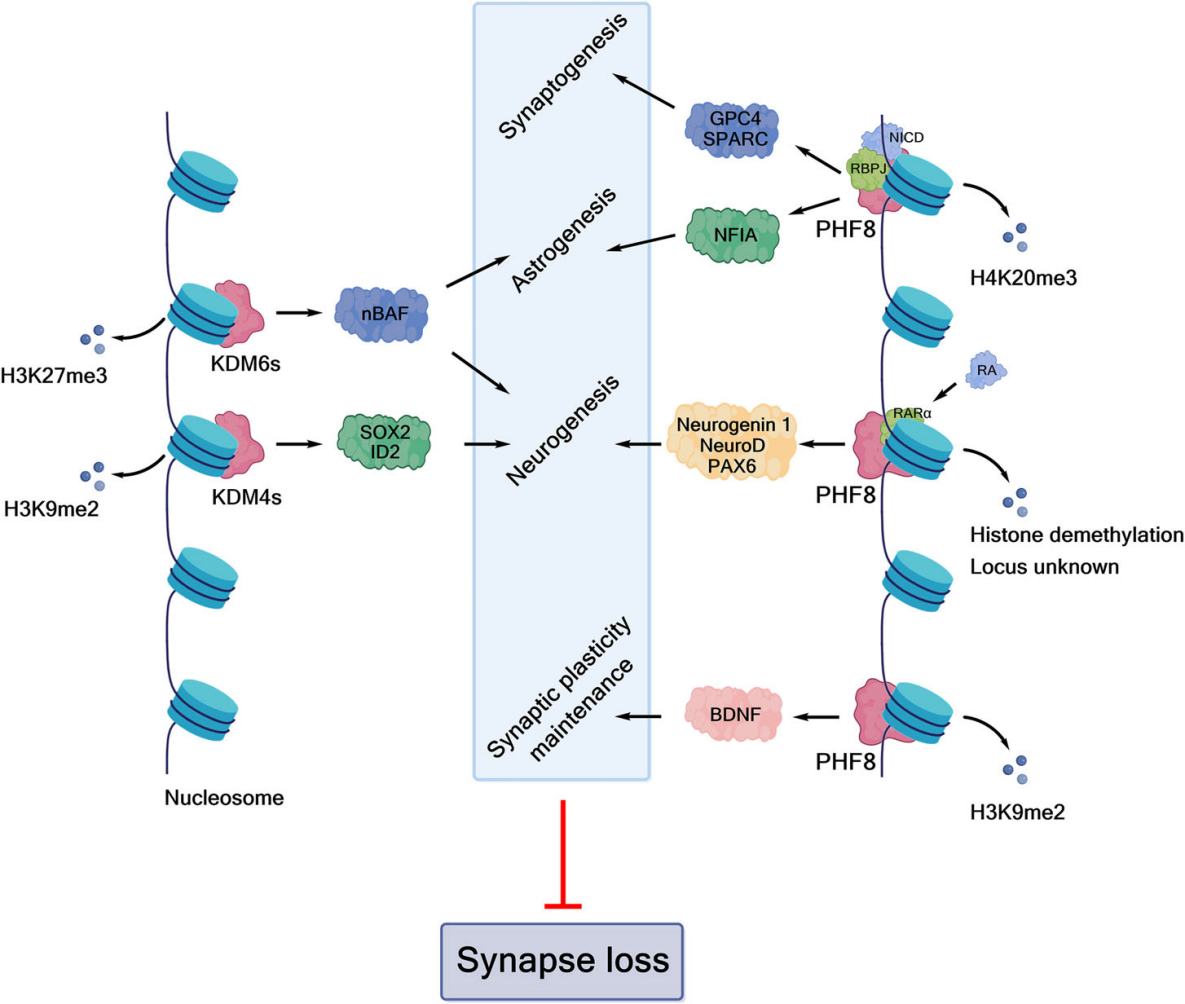
Histone demethylase‐regulated epigenetic modifications and synapse loss. Some histone demethylases (KDMs), for example KMD4, KDM6, PHF8, and potentially KDM5, could regulate neuronal/glial differentiation through enzyme activity‐dependent mechanisms, thus contributing to resistance of synapse loss. (The figure was created with biorender.com.)

Other members of the structural subfamily of 2OGDD to which FIH (but not the PHDs) belongs, that is the Jumonji C (JmjC) 2OGDD, have been shown to be N^e^‐methyl‐lysine histone demethylases (KDMs).^14^ Together with other assignments of 2OGDD function in the regulation of protein biosynthesis, including the identification of 2OGDD catalyzing modifications of nucleic acids such as the ten eleven translocation (TET) 2OGDD, this work has revealed very broad roles for 2OGDD, and as a consequence the direct reaction with oxygen, in the modification of macromolecules involved in the regulation of expression/protein biosynthesis.^15^ These findings raise the question as to whether changes to chromatin in AD patients leading to altered synaptic function are linked to the normal or impaired activities of one or more of the 60 to 70 human 2OGDD. There is evidence that some 2OGDD (JmjC KDMs) are involved in synaptic function^16^, ^17^ and neural defects^18^ and likely others will be involved (along with many other factors). We propose that modulation of JmjC KDM activity could rescue synapse loss through regulating astrogliogenesis, synaptogenesis, or neurogenesis (Figure 2).

### 1.3 Study limitations and perspective

The complexity and context‐dependent natures of the roles of many 2OGDD, especially those involved in the regulation of transcription, such as the JmjC KDMs, means work to identify preferred targets among them for AD treatment is challenging. Presently, there is no direct evidence of role for 2OGDD in AD development in the manner that there is for them in some cancers (e.g., the TETs and certain JmjC KDMs).^19^ However, as we have described below there are interesting links between AD and some 2OGDD (e.g., PHYD1) and cases can be made for the involvement of multiple 2OGDD in AD progression. These links need to be validated by systematic studies on 2OGDD and AD progression, with a focus on the hippocampus, which is the part of the brain most damaged by AD. Given the complexity of the roles of 2OGDD in human biology we believe such work is best done in a highly collaborative and interdisciplinary manner. The research should range from genetic and clinical studies to molecular biology, using both resources for pathology (e.g., the China Human Brain Bank Consortium^20^, ^21^) and advanced methods for modeling AD, for example, using human induced pluripotent stem cell (iPSC)‐derived brain cells to explore synapse loss.^22^, ^23^ There is also potential for measurement of 2OGDD or their reaction products to be used in diagnosis of types of AD, both in patients and in pathological studies. The collagen biosynthesis 2OGDD PLOD1 is a potential prognostic blood biomarker of early AD^24^ and investigation of other 2OGDD acting on blood proteins (e.g., aspartate/asparagine‐β‐hydroxylase [ASPH]) is of interest from a biomarker perspective. To identify novel therapeutic targets, an unbiased genetic screening for the involvement of 2OGDD is recommended.^25^

Pioneering work with prolyl hydroxylase inhibitors has identified mechanisms of inhibiting 2OGDD via 2OG competition/active site Fe(II) chelation; although other types of 2OGDD inhibition can be envisaged, developing brain penetrating versions of active site targeting inhibitors may be a good place to start such work on exploring the utility of 2OGDD inhibition outside of the hypoxic response in AD treatment. The recent approval of PHD inhibitors for treatment of anemia in chronic disease means that at least some of the human 2OGDD are therapeutically tractable and as far as is known safe targets. However, the current clinically approved PHD inhibitors are optimized for treatment of anemia in chronic kidney disease via upregulation of EPO production in the liver and kidneys. While these drugs might be of some value in treating the symptoms of AD, they are not optimized for brain penetration; in fact, this was likely minimized during their development. We therefore propose that development of brain penetrating PHD inhibitors, with and without additional activity versus FIH (which contributes to regulation of the set of HIF target genes), is pursued with a view to investigating their potential for AD treatment, via alteration of synaptic function in the hippocampus, either mediated via EPO upregulation or not.

It should be[Fig alz12733-fig-0003] noted that the therapeutic manipulation of 2OGDD for AD treatment might be related to a direct role of abnormal 2OGDD activity in the development of AD, but this is not necessarily so. There is evidence that the sets of HIF target genes upregulated in response to hypoxia or hypoxia mimics is context dependent.^26^ Further, although in effect the PHD inhibitors act as hypoxia mimics, in vitro work implies that their precise effects might not mimic those of natural hypoxia.^27^ There is thus interest in exploring potential AD treatments involving PHD inhibition combined with manipulation of other aspects of “epigenetic” transcriptional regulation, to alter expression patterns of HIF target genes, including those not normally regulated by HIF. Manipulation of the set of genes upregulated by HIF may include modulation of the activity of other 2OGDD, for example, FIH or JmjC KDMs, but is not limited to 2OGDD. One potential target is to promote APP and so hinder synapse loss, which is fundamental to the pathophysiology of AD.^4^

Below we give an overview of human 2OGDD and critically discuss their potential roles in AD, highlighting possibilities for future studies and clinical applications, with a particular emphasis on synapse dysfunction/loss associated with neurodegenerative disorders. We propose future research directions and methods that can be applied to test the roles of 2OGDD in AD and their suitability as drug targets. Our aims are twofold: first, we aim to promote and enable long‐term research on the roles of 2OGDD and, more generally, oxygen/hypoxia; second, we aim to encourage shorter term empirically guided clinical studies concerning 2OGDD inhibitors and other brain oxygen availability modulators in AD treatment.

## 2 CONSOLIDATED RESULTS AND IMPLICATIONS

In humans there are 60 to 70 2OGDD, the reported reactions of which comprise hydroxylations and related two electron oxidations and *N*‐methyl demethylations, the latter proceeding via initial hydroxylation.^15^, ^28^ 2OGDD act on substrates including amino acids/other small molecules, lipids, proteins, and nucleic acids. Most studied 2OGDD use Fe(II) as a cofactor and couple the oxidation of their substrates with conversion of 2OG and O_2_ into succinate and CO_2_.^15^ The activities of some 2OGDD are promoted by reducing agents, in particular catalysis by prolyl‐hydroxylases involved in collagen biosynthesis is promoted by vitamin C (*L*‐ascorbic acid), a deficiency of which causes the disease scurvy, which is associated with defective collagen (see below).

**FIGURE 3 alz12733-fig-0003:**
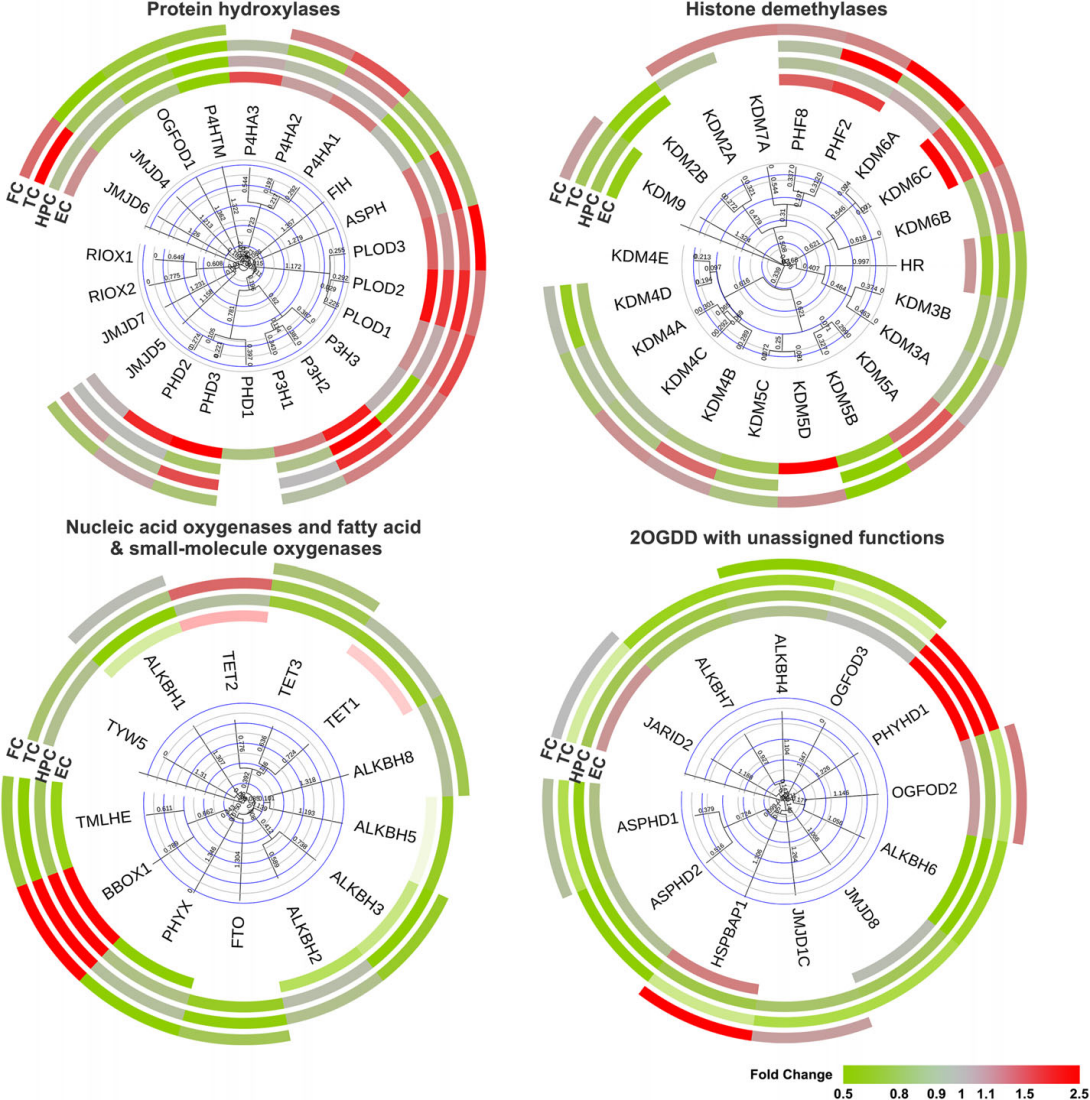
Expression profiles of assigned 2OGDD in AD brains. 2OGDD were classified according to their functions and expression levels in four brain regions of AD patient‐derived versus control samples (upregulation in red; downregulation in green). Evidence for general 2OGDD downregulation was observed, especially of 2OGDD in the ALKBH subfamily that perform nucleic acid modifications, implying that 2OGDD may play roles in epigenetic changes associated with AD progression. The results suggest that 2OGDD (i.e., PLODs) involved in collagen formation may be upregulated in AD brains, potentially impacting the extracellular matrix. The data come from AlzData (http://www.alzdata.org/), a high‐throughput collection of AD data. See Tables 1‐5 for acronym definitions and functions of human 2OGDD. 2OGDD, 2‐oxoglutarate and ferrous iron‐dependent oxygenases; AD, Alzheimer's disease; EC, entorhinal cortex; FC, frontal cortex; HPC, hippocampus; TC, temporal cortex.

Analysis of the expression profiles of 2OGDD in four brain regions, the entorhinal cortex (EC), the hippocampus (HPC), the temporal cortex (TC), and the frontal cortex (FC) suggests a link between 2OGDD and AD (Figure 3, data from AlzData [http://www.alzdata.org/], a high‐throughput collection of AD data^29^). A general downregulation of 2OGDD, especially those acting on nucleic acids, is observed in AD; the upregulation of 2OGDD involved in collagen biosynthesis in AD suggests an impact by them on the brain extracellular matrix (ECM), as reviewed earlier.^30^ The potential roles of 2OGDD in the regulation of aging processes have also been reviewed.^31^ Impaired 2OGDD catalysis may occur as a consequence of mutation/altered expression levels or localization, or as a consequence of depleted availability of substrate(s); Fe(II); 2OG; or, of particular interest to us, atmospherically derived oxygen. We propose that dysfunction of 2OGDD activity may contribute to the transformation from normal aging to neurodegenerative disease.

Pronounced cognitive decline is the major symptom that distinguishes normal aging and AD. At the pathological level in AD the massive loss of synapses strongly correlates with cognitive decline.^32^, ^33^ In addition to aging, a combination of genetic and environmental factors is also likely involved in AD progression. Tobacco smoking and diabetes, both of which are associated with hypoxia, have also been shown to be associated with substantial changes in the transcription of AD associated genes, for example, *APP* and *MAPT*.^34^ Patients with cardiovascular disease are more prone to AD^35^, ^36^ and there is abundant evidence that an inadequate supply of oxygen can affect neuronal development.^37^, ^38^ It is also known that an acute hypoxia decreases synaptic transmission and that extended hypoxia results in morphological changes in presynaptic terminals and ultimately death of neurons.^39^, ^40^ Hypoxia can also induce upregulation of APP^41^ and Aβ^42^ in vitro, although this effect might be less pronounced in vivo.^43^ However, short‐term exposure to mild/intermittent hypoxia is reported to limit damage caused by some brain injuries.^44^, ^45^, ^46^

It is perhaps obvious, but worth emphasizing, that atmospherically derived oxygen is a prime source of energy, including via enabling ATP production, for all aerobic organisms including humans. Maintaining oxygen homeostasis is thus important for *all* aspects of human/animal biology and especially so for the brain which, along with the heart, is acutely sensitive to oxygen availability.^47^ Oxygen supply therefore likely plays a key role in the development of most, if not all, brain‐related diseases including dementia/AD. There are emerging clinical links between hypoxia and AD. For example, a pilot study has indicated that levels of tau protein in blood and cerebrospinal fluid (CSF) increase after myocardial infarction.^48^ In aged patients with acutely decreased blood pressure, reduced oxygen availability correlates with elevated levels of phosphorylated tau protein and memory defects.^6^, ^49^ An important objective of this article is to argue that efforts should be made to explore manipulation of the natural response to the challenge of hypoxia for AD treatment. Research on how animals respond to hypoxia extends back more than a century, but over the last 25 or so years it has been spurred on by advances in our understanding of the molecular details of how cells adapt to limiting oxygen.^7^

Compared to classical neurodegenerative pathology, for example, Aβ plaques and neurofibrillary tangles (NFTs), the role of vascular pathology in the loss of synapses in cognitive impairment in AD has perhaps been overlooked. However, vascular dysfunction and damage have been identified as critical components of the pathophysiology of late‐life dementia including AD.^50^ Vascular‐mediated pathophysiology in vascular cognitive impairment and dementia (VCID), a non‐Alzheimer's dementia, is linked to hypoxia.^35^ Hence, exploring the roles of hypoxia‐induced VCID in cognitive decline and synapse dysfunction is of interest in AD. Given 2OGDD play key roles in the natural response to hypoxia, including via regulation of VEGF levels, they are of interest from basic science and therapeutic perspectives in terms of the vascular pathology of AD.

**FIGURE 4:**
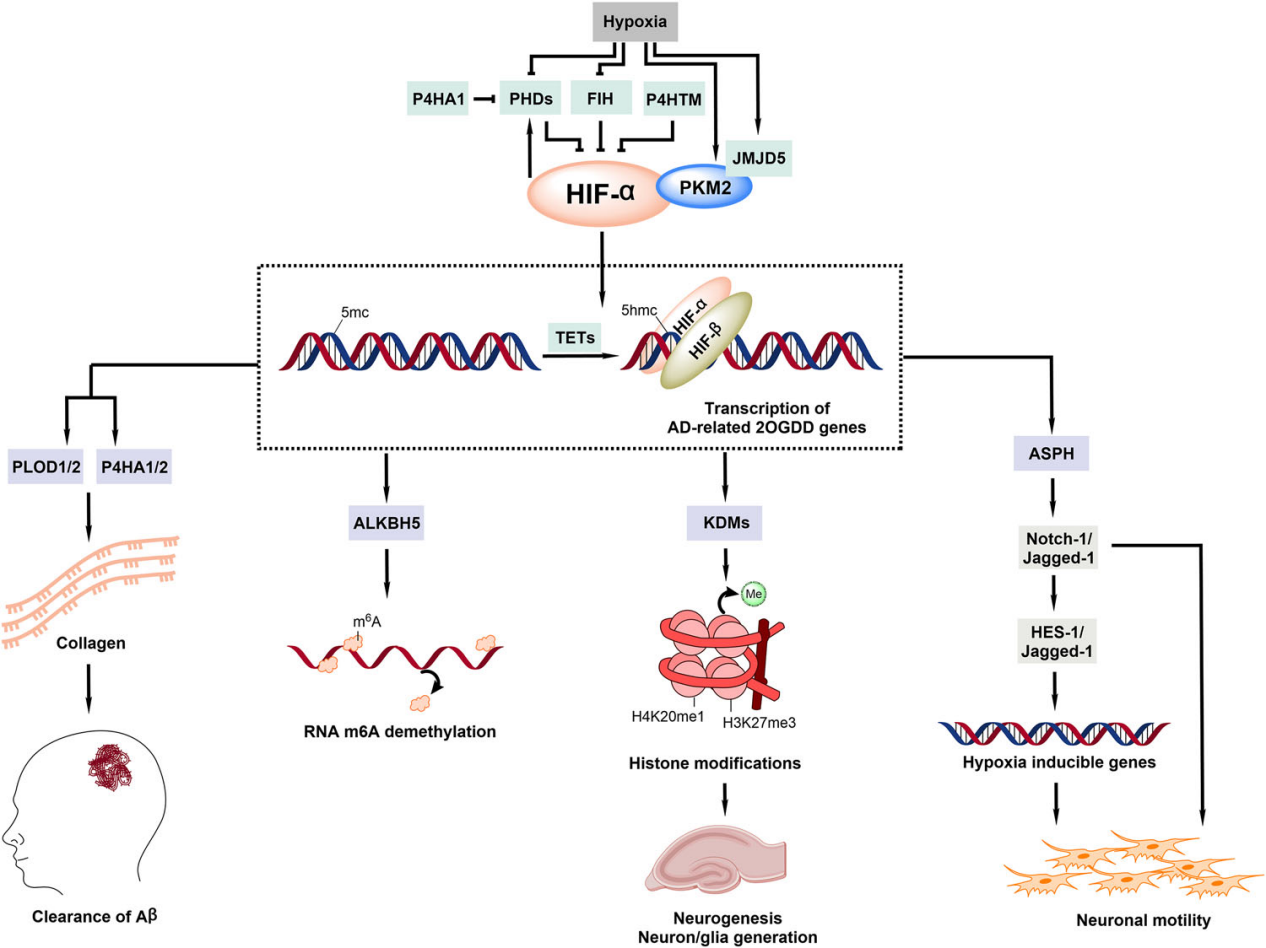
Potential roles of HIF modifying and other 2OGDD in AD. HIFs are α, β‐transcription factors that are involved in adaptation to hypoxia. There is evidence that HIF, in particular HIF‐1α, has a potential neuroprotective role in AD. Light green boxes indicate 2OGDD known/proposed to regulate HIF (the PHDs/FIH are best validated). Under hypoxia, hydroxylation by PHDs is reduced due to decreased O_2_ availability resulting in HIF‐α accumulation, dimerization with HIF‐β and migration to the nucleus, where HIF‐α/β regulate the expression of multiple genes, which work to ameliorate the effects of hypoxia. Some HIF target genes are altered in AD. FIH catalysis negatively regulates HIF activity. P4HTM is also reported to prolyl‐hydroxylate HIF‐α. It is proposed that hypoxia‐induced upregulation of JMJD5 and PKM2 results in increased abundance of the JMJD5/PKM2/HIFα complex, which could indirectly enhance HIF activity. The TETs catalyze oxidation of 5‐methylcytosine—there is potential for the TETs to regulate HIF target gene expression by altering the binding of HIFs to their target genes, which may contribute to the pathogenesis of AD. The light purple boxes show selected 2OGDD that are (proposed) HIF‐target genes. 2OGDD, 2‐oxoglutarate and ferrous iron‐dependent oxygenases; AD, Alzheimer's diseaseFIH, factor‐inhibiting hypoxia‐inducible transcription factors; HIF, hypoxia‐inducible transcription factors; PHD, prolyl‐hydroxylases; TET, ten eleven translocation

Many reported studies on 2OGDD and AD are related to the HIFs, which are α, β‐heterodimeric transcription factors, the gene targets of which, including some 2OGDD,^9^, ^51^ work to ameliorate the effects of hypoxia, that is, an inadequate supply of atmospherically derived oxygen. The 2OGDD involved in the hypoxic response are prime candidates among 2OGDD for investigation as potential targets for AD treatment (Figure 4). Both VEGF and EPO are HIF target genes and established neuroprotective factors that have a demonstrated capacity to resist synapse loss. Two different types of 2OGDD, that is FIH and PHD enzymes, catalyze hydroxylation of HIF‐α isoforms, of which there are three in humans. The PHDs and FIH play key roles in HIF regulation and both are of interest as AD therapeutic targets.

Over 20 human 2OGDD, in particular, but not exclusively, from the JmjC KDM and the TETs (which catalyze 5‐methylcytosine oxidation) 2OGDD subfamilies participate in both the healthy regulation and disease‐associated changes in transcription.^52^ Targeting disease‐associated “epigenetic” changes involving modifications to histones and DNA/RNA^15^ is an attractive option for the treatment of AD. Thus, 2OGDD involved in the regulation of transcription are likely to be of increasing interest in the study of epigenetic changes associated with AD. Importantly, several recent studies have shown that some KDMs have the potential to regulate neuronal/glial differentiation through enzyme activity‐dependent mechanisms, which may contribute to synaptic formation and connectivity.

Below we describe the functions of specific human 2OGDD focusing on (potential) links to AD, especially those that are related to synapse function; to guide the reader, we have summarized the domain organization and substrates of human 2OGDD in Tables S1–S5 in supporting information.

## 3 LINKS BETWEEN 2OGDD AND AD

### 3.1 2OGDD cosubstrate and cofactor links to AD

2OGDD use the tricarboxylic acid (TCA) intermediate 2OG and oxygen as cosubstrates and produce succinate, also a TCA intermediate, and carbon dioxide as coproducts. Recently, 2OG was observed to be significantly increased in plasma and CSF of AD patients.^54^ Further investigation found that 2OG is an antioxidant that specifically detoxifies reactive oxygen species with the concomitant formation of succinate to combat oxidative stress in AD.^55^

A reducing agent, typically but not necessarily *L*‐ascorbic acid, is often added to promote 2OGDD catalysis in vitro. Ascorbate is a pivotal antioxidant that supports neuronal health and may play a role in slowing the progression of AD.^56^ The in vivo evidence for a direct role of *L*‐ascorbic acid in 2OGDD catalysis is lacking though there is indirect evidence that the activity of some 2OGDD (prolyl hydroxylases) involved in collagen biosynthesis is promoted by *L*‐ascorbic acid. Despite this incomplete knowledge, it is plausible that 2OGDD catalysis is limited by reducing agents’, including *L*‐ascorbic acid, availability in cells. AD patients are reported to be deficient in even ascorbate, despite adequate supplementation,^57^ an observation that may result in impaired 2OGDD catalysis.

To date they have all been found to use Fe(II) as a cofactor and there is good evidence that the activity of some 2OGDD can be limited in cells by iron availability.^19^, ^53^ There is evidence indicating that iron dishomeostasis is involved in AD, including in Aβ plaque and tau pathologies.^58^ A significant elevation in iron levels in neurons has been observed in the preclinical stage of AD, especially those in the cortex and hippocampus, the main brain area affected by AD.^59^ The increased iron levels could cause perturbation of redox homeostasis and hence 2OGDD disfunction in AD, for example by promoting conversion of catalytically active Fe(II) to inactive Fe(III). Ferroptosis, an iron‐dependent form of cell death, is characterized by catastrophic iron‐induced lipid peroxidation, ultimately leading to membrane rupture.^60^ Although studies on the roles of ferroptosis in AD are at an early stage, ferroptosis has been observed in neurodegenerative diseases.^61^ Ayton et al. have proposed a role for a ferroptosis hypothesis in AD based on large cohort studies,^62^ which could be independent of the classic Aβ/tau hypothesis. Connections between ferroptosis and 2OGDD activity are emerging. Rroji et al. have shown that PHD inhibition can hinder neuronal ferroptosis so potentially enabling neuroprotection.^63^ The human 2OGDD phytanoyl CoA hydroxylase (PHYH), γ‐butyrobetaine hydroxylase (BBOX1), and trimethyl lysine hydroxylase (TMLHE) are key enzymes in lipid metabolism. PHYH is reported to be a ferroptosis‐related biomarker in kidney ischemia‐reperfusion injury and inhibiting PHYH might protect the kidney from ferroptosis.^64^ In summary, given the central importance of iron in both 2OGDD catalysis and ferroptosis, exploring their potentially linked roles in AD is of considerable interest.

### 3.2 The PHD/FIH‐HIF pathway: A potential double‐edged sword in AD

Both the levels and transcriptional activity of HIF are regulated by 2OGDD. PHD catalyzed prolyl‐hydroxylation in the *N*‐ and the *C*‐terminal oxygen‐dependent degradation domains of HIF‐α subunits signals for proteasomal degradation of HIF‐α isoforms by promoting their binding to the von‐Hippel–Lindau (VHL) protein, which is the targeting component of a ubiquitin ligase complex.^9^ The transcriptional activity of the HIF is inhibited by FIH catalyzed hydroxylation of an asparaginyl residue in the carboxy‐terminal transcriptional activation domain (CAD) of HIF‐α isoforms, a modification that hinders binding of transcriptional coactivator proteins (CBP/p300) recruitment,^9^ so modulating HIF activity in a context‐dependent manner. PHD and (to a lesser extent) FIH catalysis is suppressed by hypoxia, so enabling formation of a transcriptionally active HIF‐α, β complex.

There are three human PHD isoforms (PHD1–3, of which PHD2 is the most conserved^65^), all of which negatively regulate HIF activity,^66^, ^67^ but only one FIH isoform. There are three HIF‐α isoforms, with HIF‐1α and 2α being the best characterized—they have distinct targets for transcription, though the mechanisms by which regulation of specific sets of HIF target genes is achieved in different contexts is unclear. There is some evidence that specific PHD isoforms act preferentially on HIF‐2α or HIF‐1α.^68^ It is also unclear whether the PHDs have non‐HIF‐α substrates, though FIH has multiple other substrates, many from the ankyrin repeat domain family.^69^, ^70^ Overall, the combined studies with cells, animal models, genetic diseases, and clinically used small‐molecule inhibitors support the roles of HIF in the hypoxic response, in particular in regulation of the *EPO* and *VEGF* genes, and of the role of the PHDs as hypoxia sensors for the HIF system.^71^

Hypoxia acts as an important environmental risk factor in the onset and development of AD and there are emerging links between HIF/the PHDs and AD. HIF‐1α, a key factor in response to hypoxic stress, is considered a potential medicinal target for AD.^72^ Recent evidence of a role for HIF‐1α in Aβ pathology, neuroinflammation, and tau hyperphosphorylation has been described.^72^ Below, we focus on associations between the PHD/FIH‐HIF pathway and synapses (Table 1).

**TABLE 1:** Summary of selected roles of the HIF hydroxylases PHD 1‐3 and FIH, data on their expression and potential roles in AD

2OGDD name	Abbreviation	Altered expression in AD	Cell lines/models/samples	Known and potential functions relating to AD	Enzymatic activity	Refs.
Egl nine homologue 1 (prolyl hydroxylase domain containing 2)	EGLN1 (PHD2)		Neuron‐specific *phd2* knockout mice	Regulation of hypoxic response Promising targets for treatment of AD, inducing via regulating HIFα degradation/HIF target genes relating to neuroprotective factors (e.g., EPO, VEGF)	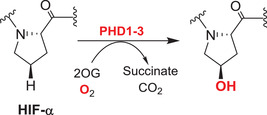	^67^
Egl nine homologue 2 (prolyl hydroxylase domain containing 1)	EGLN2 (PHD1)		PHD1‐deficient mice			^73^
Egl nine homologue 3 (prolyl hydroxylase domain containing 3)	EGLN3 (PHD3)	Upregulation in microglia	APP/PS1 mice	PHD3 could restrict Aβ phagocytosis of m icroglia, influencing soluble Aβ generation		^75^, ^77^
Factor inhibiting HIF	FIH			Regulation of hypoxic response Interacts with APP indirectly, may influence soluble Aβ generation and response to the hypoxic environment in AD brains	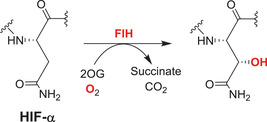	^80^, ^81^

*Note*: Table is not comprehensive both with respect to the reactions and roles of the 2OGDD.Abbreviations: Aβ, amyloid beta; AD, Alzheimer's disease; APP, amyloid precursor protein; EPO, erythropoietin; HIF‐α, the α‐subunits of hypoxia‐inducible factors; PS1, presenilin 1; VEGF, vascular endothelial‐derived growth factor; 2OG, 2‐oxoglutarate.

Studies with PHD1‐deficient mice demonstrate that *PHD*1 depletion hinders HIF‐1α degradation in hyperoxia, so promoting expression of *VEGF*, a HIF target gene and a neuroprotective factor,^73^ suggesting that PHD1 inhibition may be beneficial for neurodegenerative disorders, such as AD.^73^ Meta‐analysis of four genome‐wide association studies (GWAS) showed that *PHD1* belongs to one of the top 5% of AD‐associated genes.^74^ Neuronal PHD2 deficiency is reported to improve cognitive abilities in a murine model of cerebral hypoperfusion, along with enhanced hippocampal EPO and VEGF mRNA levels.^67^ PHD2 and PHD3 are reported to co‐regulate the transcription of EPO in brain pericytes, via a HIF‐2α dependent mechanism.^75^ EPO and VEGF are reported neuroprotective factors. EPO could drive neuroplasticity and neurogenesis via EPO/EPOR signaling.^13^ VEGF is reported to counteract toxic‐soluble Aβo‐induced synaptic dysfunction^12^ (many studies suggests that Aβo induces synapse loss in AD, rather than the Aβ plaque load^76^). Thus, although further target validation is required PHD inhibition is worthy of consideration as an AD target.

Research on PHD3 and AD microglia implies connections between the PHDs and synapse loss. *phd3*‐expressing microglia are almost exclusively restricted to amyloid plaques in the cortical areas of *App‐Psen1* mice.^77^ PHD3 limits levels of the CD45 protein on the cell surface, the expression of which contributes to enhanced phagocytic capacity of microglia.^77^ Similarly, PHD3 restricts microglial Aβ phagocytosis and its absence reduces the total Aβ content, but increases the soluble Aβ fraction.^77^ The absence of PHD3 in microglia in AD mouse models potentiates beneficial responses in plaque‐associated microglia (PAM).^77^ It seems that during AD progression, Aβ plaques induce PHD3 expression in Aβ PAM, thus inhibiting phagocytosis, uptake, and clearance of Aβ. It is possible that the relatively high concentrations of 2‐OG and Fe(II) in AD^54^, ^78^ alter PHD activity in a manner reducing levels of HIF‐α targeting neuroprotective factors. The combined research highlights a need for detailed explorations on the roles of PHDs and their modulation in the Aβo/microglia‐mediated synapse loss during progression of AD.

Studies on FIH and AD are at an earlier stage than for the PHDs and are complicated by the multiple FIH substrates.^79^ It is reported that Aβ A4 precursor protein‐binding family A member 3 (APBA3) enhances HIF‐1α expression in macrophages by binding to FIH via the *N*‐terminal domain of APBA3 so regulating macrophage‐mediated inflammation.^80^ APBA3 binds APP via its *C*‐terminal domain and may modulate processing of APP and hence, formation of Aβ.^81^ Thus, APBA3 may act as an adaptor in recruiting cytoplasmic FIH to sites where APBA3 is localized, that is it may mediate indirect association of FIH with APP,^80^ so contributing to generation of soluble Aβ. It is possible that the FIH/APBA3/APP axis plays a role in Aβo/microglia‐mediated synapse loss, neuroinflammation, and Aβ accumulation in AD.

Regulation of the PHD/HIF‐1α/VEGF/EPO axis is a potential target for AD treatment.^67^, ^73^ PHD inhibition could prevent mitochondrial dysfunction and oxidative stress in AD^82^ and studies have demonstrated that small‐molecule PHD inhibitors protect neurons from oxidative injury.^83^, ^84^, ^85^ PHD inhibitors (which are 2OG competitors) targeting all three PHD isoforms are approved for the treatment of anemia in chronic kidney disease.^86^, ^87^ These are not optimized for delivery to the brain, which is not a major site of EPO production. Nonetheless, given that there is preliminary evidence that EPO treatment may have clinical benefit in treatment of mild to moderate AD,^88^, ^89^ empirically guided approaches involving boosting EPO levels/oxygen supply to the brain via use of the currently available PHD inhibitors developed for treatment of anemia by the promotion of erythropoiesis may be of interest.

Early studies provide evidence that pursuing brain penetrating PHD inhibitors is of interest with respect to treating AD. 5‐(*N*‐Methyl‐*N*‐propargyaminomethyl)‐8‐hydroxyquinoline (M30) and 5‐[4‐propargylpiper‐azin‐1‐ylmethyl]‐8‐hydroxyquinoline (HLA20) are brain permeable, iron chelating drugs, which compete for Fe(II) with the PHDs, so increasing HIF‐α levels and inducing the transcription of HIF‐α regulated neuroprotective genes. These drugs have shown pro‐cognitive and anti‐inflammatory effects in a mouse model of AD.^90^, ^91^, ^92^ Although M30 and HLA20 likely inhibit other 2OGDD, there appears to be sufficient evidence to merit systematic studies on the roles of PHD1–3 in the brain and their evaluation as AD targets, including by the use of broad spectrum and PHD isoform selective inhibitors.

It should be noted that the apparently pleiotropic nature of the HIF system means predicting the consequences of PHD inhibition in AD patients is difficult. Upregulation of HIF‐1α increases APP cleavage and Aβ generation by promoting the expression of its transcriptional targets, the β‐ and γ‐secretases.^72^ Interestingly, it is reported that in hypoxia HIF‐1α can directly bind to γ‐secretase in a manner that promotes its cleavage of Notch.^93^ Inhibition of the PHDs and/or FIH could thus exacerbate the production of Aβ, inducing synapse loss. Research involving combination of M30/HLA20/other PHD inhibitor and a β‐/γ‐secretase inhibitor may be worth exploring.

### 3.3 2OGDD histone demethylases: Potential candidates for brain repair

Post‐translational histone modifications are proposed to play roles in AD.^34^, ^94^, ^95^, ^96^ For example, H3K9me2 levels are substantially upregulated in the prefrontal cortex lysates of aged familial AD mice, P301S tau transgenic mice, and AD patients,^97^ and in the occipital cortex of post mortem AD patients.^98^ By contrast, H3K9me2 was found to be downregulated in human AD hippocampal neurons, suggesting region specificity in changes in H3K9me2 levels relating to AD.^99^ In early AD disease, an increased H3K4me3 level is reported in the cytoplasm with significant colocalization with tau markers, while the levels were decreased in the nuclei of AD brains.^100^

Because they are the largest family of KDMs, the 2OGDD dependent KDM2‐7, nine subfamilies^101^ are of interest from an AD perspective (Table 2). Unlike the flavin dependent lysine specific demethylases (LSDs, KDM1s), which only catalyze demethylation of mono‐ and di‐*N* ^e^‐methylated lysine residues, the JmjC KDMs catalyze demethylation of all three *N* ^e^‐methyl lysine methylation states. Some JmjC KDMs have *N*‐methyl arginine demethylase activity in isolated form, though the importance of this in cells is not established.^102^ The methylation state and residue selectivity of the JmjC KDMs varies; JmjC KDM activity can be transcriptionally activating or repressing depending on the residue targeted. Analysis of the roles of KDMs is challenging due to the multiplicity of histone modifications (in particular of the histone H3 tail), which are of central importance in regulation of eukaryotic transcription. The JmjC KDMs are multidomain proteins and their activities are likely regulated, at least in part, by interactions away from their catalytic domains as nicely exemplified with regulation of the KDM activities of PHF8 (KDM7B) and KIA1718 (KDM7A) by binding of their plant homeobox domain to H3K4me3.^103^ Human JmjC KDMs have a range of activities as summarized in Table S2 and reviewed elsewhere.^101^

**TABLE 2:** Summary of selected roles of human JmjC KDMs, their expression levels and potential roles in AD

2OGDD name	Abbreviation	Altered expression in AD	Cell lines/models/samples	Known and potential functions relating to AD	Enzymatic activity	Refs.
F‐box and leucine‐rich repeat containing 11	FBXL11 (KDM2A)	Upregulation in prefrontal cortex	AD patients’ post mortem brain tissue	Involved in tauopathies	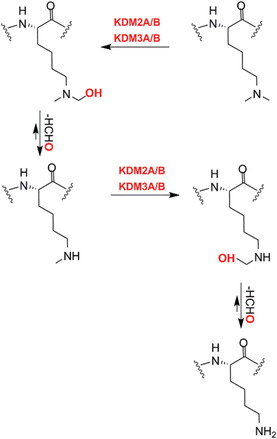 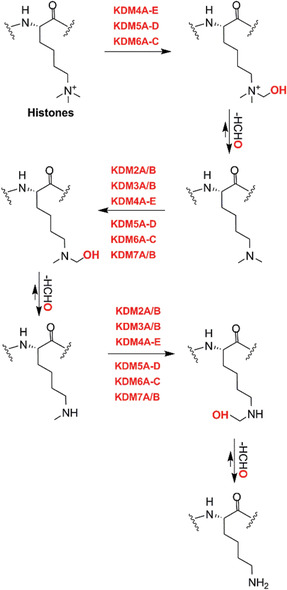	^115^
F‐box and leucine‐rich repeat containing 10	FBXL10 (KDM2B)	Downregulation in prefrontal cortex	AD patients’ post mortem brain tissue	Involved in tauopathies and interacts with PRC2, which is associated with LOAD,so modulating expression		^115^, ^136^
Jumonji domain containing 1A	JMJD1A (KDM3A)	Upregulation in prefrontal cortex	AD patients’ post mortem brain tissue	Involved in tauopathies		^115^
Jumonji domain containing 1B	JMJD1B (KDM3B)	Upregulation in prefrontal cortex	AD patients’ post mortem brain tissue	Involved in tauopathies		^115^
Jumonji domain containing 1C	JMJD1C (KDM3C)					
Jumonji domain containing 2A	JMJD2A (KDM4A)	Upregulation in prefrontal cortex	AD patients’ post mortem brain tissue	Promotes heterochromatic loss, further inducing tau‐mediated neurodegeneration		^115^
Jumonji domain containing 2B	JMJD2B (KDM4B)	Upregulation in prefrontal cortex	AD patients’ post mortem brain tissue	Involved in tauopathies		^115^
Jumonji domain containing 2C	JMJD2C (KDM4C)					
Jumonji domain containing 2D	JMJD2D (KDM4D)			Involved in hippocampal dentate gyrus (DG) neurogenesis		^107^, ^108^
Jumonji domain containing 2E	KDM4E					
Jumonji, AT rich interactive domain 1A	JARID1A (KDM5A)	Upregulation in expression level	Meta‐analysis			^116^
Jumonji, AT rich interactive domain 1B	JARID1B (KDM5B)			Interacts with PRC2, which is associated with LOAD, so modulating expression		^137^
Jumonji, AT rich interactive domain 1C	JARID1C (KDM5C)			Regulates neuronal transcriptional programs for proper neurodevelopmental process and synaptic structure and function		^16^, ^17^, ^109^
Jumonji, AT rich interactive domain 1D	JARID1D (KDM5D)					
Ubiquitously transcribed tetratricopeptide repeat containing X chromosome	UTX (KDM6A)			Required for long‐term proliferation human neural progenitor cells (hNPCs) and further generation of neurons/glia		^273^
Ubiquitously transcribed tetratricopeptide repeat containing Y chromosome	UTY (KDM6C)				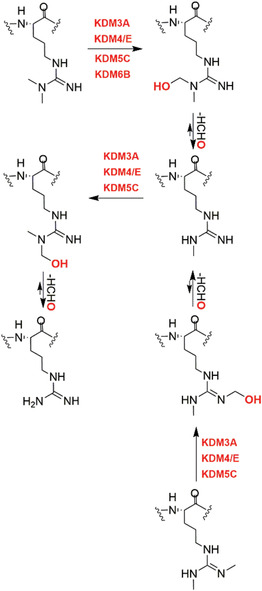	
Jumonji domain containing 3	JMJD3 (KDM6B)		Mouse NSC line	Regulates neural‐specific expression via interacting with TAp63γ; Required for long‐term proliferation of human neural progenitor cells (hNPCs) and further generation of neurons/glia.		^117^, ^273^, ^274^
PHD finger containing 2	PHF2					
PHD finger containing 8	PHF8		Aluminum‐Exposed Rat Model Mouse NSCs	Prevents synaptic plasticity injury; proposed key regulator of astrogliogenesis and synaptogenesis		^113^, ^114^
Jumonji C domain containing histone demethylase 1 homologue D	JHDM1D (KDM7A)					
Lysine‐specific demethylase hairless	HR				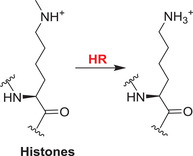	
Jumonji domain containing 4	JMJD4				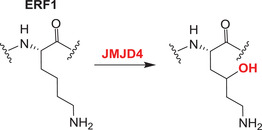	
Jumonji domain containing 5	JMJD5			Potentially regulates HIF‐1α transactivation activity Involved in Aβ‐inducible neuroinflammation	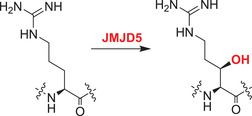	^161^, ^162^, ^163^ ^,^ ^165^, ^275^
Jumonji domain containing 6	JMJD6				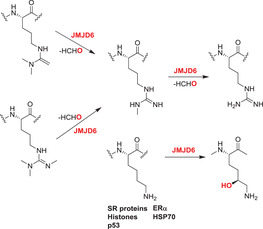	^276^, ^277^, ^278^
Jumonji domain containing 7	JMJD7			Affects phosphorylation of tau	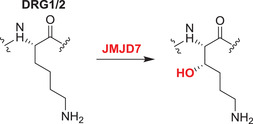	^144^, ^166^
Jumonji domain containing 8	JMJD8			Regulates HIF‐1α transactivation activity, involved in neuroinflammation		^167^, ^168^
HSPB (heat shock 27kDa) associated protein 1	HSPBAP1		AD post mortem brains	Influences neuroprotective function of HSPB1		^170^, ^172^
Chromosome 2 open reading frame 60 (tRNA‐wybutosine synthesizing enzyme 5)	C2orf60 (TYW5)				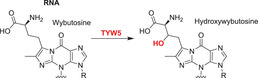	
Mineral dust induced nuclear antigen with a molecular mass of 53 kDa (ribosomal oxygenase 2)	MINA53 (RIOX2)				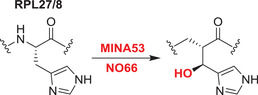	
Chromosome 14 open reading frame 169 (Nucleolar protein 66, ribosomal oxygenase 1)	C14orf169 (NO66, RIOX1)					
Jumonji, AT rich interactive domain 2	JARID2			Acts as an accessory protein of the PRC2 complex, which is associated with LOAD, so modulating expression		^128^, ^135^

*Note*: Table is not comprehensive both with respect to the reactions and roles of the JmjC 2OGDD. Abbreviations: AD, Alzheimer's disease; ERα, estrogen receptor alpha; HIF‐1α, the α subunit of hypoxia‐inducible factor‐1; HSP70, heat‐shock protein 70 kDa; JMJD, Jumonji C (JmjC) domain‐containing; KDM, *N^ε^*‐methyl‐lysine demethylase; LOAD, late‐onset Alzheimer's disease; NSC, neural stem cell; PRC2, polycomb repressive complex 2; RPL27/28, ribosomal protein L27/28; SR, splicing regulatory; TAp63γ, the full‐length isoform of p63, containing a transactivation (TA) domainAl, aluminum.

#### 3.3.1 JmjC KDMs

In general, neurogenesis appears to be a sequential and highly ordered process involving cell lineages ranging from PSCs, to neural progenitor cells (NPCs), to various subtypes of neurons and glial cells.^104^, ^105^, ^106^ Definitive links between specific JmjC KDMs and AD pathology are lacking, but their validated roles in transcription mean they are of considerable interest. Recent studies have shown JmjC KDMs have roles in neurogenesis, especially neuronal and astrocyte differentiation, potentially via regulating expression of genes involved in neuronal/glial differentiation in a demethylase activity‐dependent manner, by demethylating repressive epigenetic marks, such as H3K9, H3K27, and H4K20.^16^, ^17^, ^107^, ^108^, ^109^, ^110^, ^111^, ^112^, ^113^, ^114^ We speculate that modulation of JmjC KDM activity could rescue synapse loss through regulating astrogliogenesis, synaptogenesis, or neurogenesis.

KDM4D has a role in hippocampal dentate gyrus (DG) neurogenesis, which drops sharply in AD patients.^107^, ^108^ The JmjC KDM KDM5s, especially KDM5C, regulate neuronal transcriptional programs for proper neurodevelopmental process and synaptic structure and function.^16^, ^17^, ^109^ JMJD3 (KDM6B) and UTX (KDM6A) have been shown to have roles in adult neurogenesis via knockout mice studies;^110^, ^111^ PHF8 is involved in neuronal differentiation,^112^ especially in astrocyte differentiation and synapse formation.^113^ Li et al. reported that aluminum ions inhibit PHF8 via an undefined mechanism, possibly involving active site binding, hindering H3K9me2 demethylation and reducing brain‐derived neurotrophic factor (BDNF) levels, ultimately leading to synaptic plasticity injury.^114^

A study focusing on JmjC KDMs in tauopathies reports expression levels of *FBXL11 (KDM2A), JMJD1A/B (KDM3A/B)*, and *JMJD2A/B (KDM4A/B)* are substantially higher in post mortem prefrontal cortex tissue from patients with AD compared to controls, whereas *FBXL10 (KDM2B)* mRNA levels are downregulated in the AD patient brain.^115^ A meta‐analysis of AD at the mRNA level reports that *KDM3B* and *KDM5A* show a trend of upregulation in AD in 12 datasets.^116^ Knockdown of *KDM2*, *KDM3*, *KDM4A*, or *KDM4B* genes in flies overexpressing tau^R406W^ ameliorated tau^R406W^‐engendered eye defects, indicating JmjC KDMs have roles in neuronal function.^115^ Knockdown of *KDM4A* reduces heterochromatic loss in a tau^R406W^‐induced transgenic drosophila model;^115^ the level of H3K9me2, which is a KDM4A substrate, was decreased in tau transgenic drosophila heads compared to controls, consistent with the proposal that tau proteins promote neurodegeneration through global chromatin relaxation.^99^ KDM4A (and other JmjC KDMs) could thus be associated with heterochromatin alteration in tauopathies of AD by catalyzing H3K9me2 demethylation. The association between KDM6B (JMJD3) and AD has been reviewed elsewhere.^117^

The links among hypoxia, HIF, and JmjC KDMs are also of interest to AD, but are complex. It is reported that during AD progression, impaired Krebs cycle function and low‐level hypoxia alters the activity and expression of JmjC KDMs,^118^ some (including KDM2B/3A/4B/4C/5B/6B), but not all, of which are HIF target genes.^118^ Some JmjC KDMs are reported to be induced by pseudohypoxic stress, which activates HIF‐1α signaling in normoxia via oxidative stress, leading to alterations in mitochondrial metabolites, ceramide/sphingosine‐1‐phosphate, and the insulin/PI3K/mTOR, NF‐κB, JAK/STAT, and TGF‐β/Smad3 pathways,^119^ all of which are abnormal in AD.^120^, ^121^, ^122^, ^123^, ^124^, ^125^ Evidence has been presented that KDM6A^126^ and KDM5A^127^ are direct sensors of hypoxia, as may be the case with some other JmjC KDMs. Thus, the hypoxic environment in AD brains could affect JmjC activity KDMs directly or indirectly, further mediating gene regulation through changes in histone methylation levels.

#### 3.3.2 JARID2

JARID2 is a complex protein that has homology with JARID1s (KDM5s), as defined by the presence of conserved JmjC/JmjN and ARID domains. As yet, however, JARID2 has not been shown to have catalytic activity, but it is important in the regulation of transcription. The interaction of JARID2 with the polycomb repressive complex 2 (PRC2) confers transcriptional repression, at least in part, through production of H3K27me3^128^ and enabling PRC2 recruitment to genomic loci. miR‐155, one of the most well‐studied immune‐related miRNAs in AD‐associated neuroinflammatory events, is strongly upregulated in human AD brains^129^ and 3xTg AD mouse brains.^130^ JARID2 downregulation as mediated by miR‐155 overexpression has been shown in tumor cell lines,^131^ Th17 cells,^132^ and primary myelofibrosis (PMF) CD34^+^ cells.^133^ Based on analysis of the association between PRC2 and AD, it has been proposed that reduction of PRC2 in aged brains increases the risk of late‐onset AD (LOAD) development.^134^, ^135^ KDM2B increases the capacity of PRC2 to enhance histone H3 lysine 27 trimethylation (H3K27me3)^136^ and KDM5B and PRC2 interact to demethylate H3K4me2/3 and methylate H3K27 to give H3K27me3, leading to repression of transcription.^137^ Given the association between PRC2 and AD,^135^ further studies on the contribution of JmjC KDMs and JARID2 in AD are of interest.

### 3.4 JmjC “hydroxylases”: Potential regulatory factors of AD‐related proteins

In humans, the JmjC domain‐containing protein family contains ≈33 members, many with KDM activity (see above).^138^, ^139^ The ≈10 JmjC 2OGDD with (likely) non‐KDM activities typically have a lower molecular weight (<100 kDa) than the JmjC KDMs and fewer (if any) non‐catalytic domains; they are typically dimeric. They are referred to as JmjC “hydroxylases,” because most of them catalyze the formation of stable alcohol products, including via hydroxylation of asparagine‐, aspartate‐, histidine‐, lysine‐, and arginine residues as well as (in at least one case) RNA^140^, ^141^ (Table 2).

The catalytic activity of a JmjC domain enzyme was first demonstrated for FIH, which hydroxylates asparagines in HIF‐α isoforms and both asparagines and other residues in non‐HIF‐α susbtrates.^10^, ^11^ Subsequently, other JmjC hydroxylases have been identified. JMJD4 catalyzes C4‐lysine hydroxylation of eukaryotic release factor 1 (eRF1) at Lys‐63, a modification which promotes the ability of eRF1 to terminate translation.^142^ JMJD5, JMJD6, and JMJD7 are arginine (JMJD5) or lysine (JMJD6, JMJD7) hydroxylases, with JMJD5/JMJD7 also having reported endopeptidase activities (though the latter needs to be validated).^143^ ^—^ ^147^ JMJD6 is reported to have arginine demethylase activity, though this requires validation.^148^ ^—^ ^152^ NO66 (RIOX1) and MINA53 (RIOX2) are ribosomal histidine hydroxylases;^153^ RIOX1 hydroxylates the 60S ribosomal protein L8 at His‐216, while RIOX2 hydroxylates the L27a at His‐39.^153^, ^154^, ^155^ They are also reported to have KDM activities, with RIOX1 demethylating at H3K4me3/1 and H3K36me2 and RIOX2 demethylating H3K9me3.^154^, ^155^, ^156^ One JmjC hydroxylase has been shown to act on tRNA: TYW5 (C2orf60) hydroxylates 7‐(α‐amino‐α‐carboxypropyl) wyosine (yW‐72) to form undermodified hydroxywybutosine (OHyW*) during wybutosine biosynthesis.^157^ The catalytic functions of JMJD8 and HSPBAP1 are undefined. The combined results on the JmjC hydroxylases reveal that they are important in human development and that some (e.g., FIH, JMJD6), but not all, are notably promiscuous in terms of the range of substrates they oxidize and, potentially, the types of reaction they catalyze. The known and potential roles of selected JmjC “hydroxylases” in AD are described below.

#### 3.4.1 JMJD5

JMJD5 is likely essential in animals and is reported to have both KDM and, on the basis of detailed studies with peptides, arginine C‐3 hydroxylase activities.^145^, ^158^ It has been shown to play roles in the circadian systems of both plants and humans.^159^ Peptides from human regulators of chromosome condensation domain‐containing protein 1 (RCCD1) and ribosomal protein S6 (RPS6) have been shown to be substrates of isolated JMJD5 and crystallographic studies are consistent with the finding that JMJD5 is a JmjC hydroxylase rather than a KDM.^145^

JMJD5 is proposed to have multiple roles including regulating osteoclast differentiation by destabilizing nuclear factor of activated T‐cells, cytoplasmic 1 (NFATC1),^160^ a transcription factor in memory cells that is increased in AD patients.^161^, ^162^, ^163^ It is possible that interaction of JMJD5 with NFATC1 regulates its stability. Interaction of JMJD5 with pyruvate kinase M2 (PKM2) may hinder PKM2 tetramerization, which is required for its activity.^164^ The hypoxia in the tumor microenvironment may activate the JMJD5/PKM2/HIF‐1α axis, enhancing HIF‐1α transactivation activity, and paving the way for reprograming of cell metabolism.^165^ Activation of the JMJD5/PKM2/HIF1‐α axis in AD brains requires further verification.

#### 3.4.2 JMJD7

JMJD7 catalyzes (3*S*)‐lysyl hydroxylation of developmentally regulated GTP‐binding proteins 1 and 2 (DRG1/2), promoting their binding to RNA and so regulating translation (the precise biochemical roles of the DRGs in translation are unclear).^144^ DRG2 knockdown inhibits GSK3β phosphorylation, which results in reduced phosphorylation of tau and further enhances microtubule stability.^166^ Tau is hyperphosphorylated and then forms intraneuronal NFT in AD patients; it is possible that DRG2 modulates tau phosphorylation and that the progression of AD is impeded by inhibition of DRG2. The potential of the JMJD7/DRG2/GSK3β axis to regulate tau phosphorylation in the AD brain requires investigation.

#### 3.4.3 JMJD8

The catalytic function of JMJD8 is undefined, but similarly to JMJD5, an interaction between JMJD8 and PKM2 has been reported in ECs and shown to enhance metabolic activity.^167^ The role, if any, of the JMJD8/PKM2/HIF‐1α axis in AD brain requires investigation. JMJD8 is a positive regulator of tumor necrosis factor (TNF)‐induced nuclear factor (NF)‐κB signaling, which is required for TNF‐induced NF‐κB‐dependent gene expression,^168^ which plays a role in AD.^123^ Thus, the JMJD8/TNF/NF‐κB axis may play a role in neuroinflammation in AD.

#### 3.4.4 HSPBAP1

The heat shock protein (HSPB1)‐associated protein 1 (HSPBAP1) is a likely 2OGDD, the enzymatic activity of which is unknown, but which is reported to interact with the HSPB1 and so suppress the ability of the latter to protect cells from heat shock.^169^ The potential roles of HSPBAP1 in the brain have been investigated. In the anterior temporal neocortex of intractable epilepsy patients, HSPBAP1 mRNA levels are significantly upregulated; HSPBAP1 may contribute to disease development by disabling the known neuroprotective function of HSPB1.^170^ Whether HSPBAP1 plays a similar role in AD is unclear. MiR‐455‐3p, which is significantly upregulated in AD brains, appears to be involved in AD progression by binding its target gene, *HSPBAP1*.^171^ As a member of the small heat shock protein (sHuSP) family, HSPB1 may prevent aggregation of partially unfolded proteins,^172^ including Aβ^173^ and tau.^174^ Thus, the miR‐455‐3p/HSPBAP1/HSPB1 axis could contribute to aggregation of Aβ and tau in AD. It is proposed that HSPBAP1 is related to the response to alcohol in the nervous system.^175^ Observational studies indicate that light‐to‐moderate alcohol consumption reduces the risk of AD; however, two‐sample Mendelian randomization (MR) analysis show a causal association between alcohol consumption and an earlier AD age of onset survival (AAOS).^176^ The potential relationships among alcohol, HSPBAP1 and AD require clarification.

### 3.5 2OGDD involved in lipid metabolism: Possible mediators of lipid disorder in AD

#### 3.5.1 BBOX1/TMLHE

2OGDD play key roles in lipid metabolism in humans (Table 3). Carnitine, which is required for fatty acid transport into mitochondria, is synthesized endogenously and obtained from the diet.^177^, ^178^ 2OGDD catalyze two hydroxylation steps in carnitine biosynthesis, that is, *N^ε^*‐trimethyl‐lysine hydroxylase (TMLH) and γ‐butyrobetaine hydroixylase (GBBH) catalyze the C‐3 hydroxylation of *N^ε^*‐trimethyl‐lysine and γ‐butyrobetaine, respectively.^177^

**TABLE 3:** Human 2OGDD involved in lipid metabolism and collagen biosynthesis, their expression levels and potential roles in AD

2OGDD name	Abbreviation	Altered expression in AD	Cell lines/models/samples	Known and potential functions relating to AD	Enzymatic activity	Refs.
Trimethyllysine hydroxylase, epsilon	TMLHE			Biosynthesis of carnitine (first step), which is diminished in AD brains	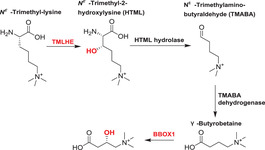	^179^, ^279^
Butyrobetaine (gamma), 2OG dioxygenase 1 (γ‐butyrobetaine hydroxylase)	BBOX1 (GBBH)	Upregulation in expression level	Meta‐analysis	Biosynthesis of carnitine (final step), which is diminished in AD brains		^116^, ^179^, ^279^
Phytanoyl‐CoA 2‐hydroxylase	PHYH (PAHX)			Phytanic acid metabolism Involved in peroxisomal dysfunction linked to Aβ production and tau phosphorylation	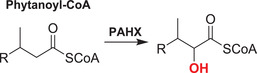	^184^, ^185^, ^186^, ^187^, ^188^
Pro‐collagen lysyl oxygenase domain 1	PLOD1	Upregulation in whole blood	Whole blood samples	Biosynthesis of collagens Associated with progression in early stage AD	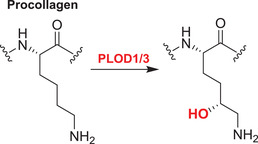	^24^, ^200^
Pro‐collagen lysyl oxygenase domain 2	PLOD2			Biosynthesis of collagens		
Pro‐collagen lysyl oxygenase domain 3	PLOD3	Upregulation in expression level	Meta‐analysis			^116^
Leucine proline‐enriched proteoglycan (Leprecan, Prolyl 3‐hydroxylase 1)	LEPRE1 (P3H1)	AD‐related gene	Human serum samples		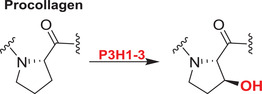	^203^
Leprecan‐like 1 (Prolyl 3‐hydroxylase 2)	LEPREL1 (P3H2)	AD‐related gene	Human serum samples			^203^
Leprecan‐like 2 (Prolyl 3‐hydroxylase 3)	LEPREL2 (P3H3)	AD‐related gene	Human serum samples			^203^
Prolyl 4‐hydroxylase subunit alpha‐1	P4HA1	AD‐related gene	GWAS meta‐analysis		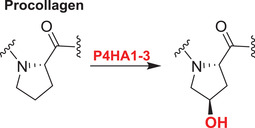	^74^, ^201^
Prolyl 4‐hydroxylase subunit alpha‐2	P4HA2					
Prolyl 4‐hydroxylase subunit alpha‐3	P4HA3					

*Note*: Table is not comprehensive both with respect to the reactions and roles of the 2OGDD. Abbreviations: AD, Alzheimer's disease; GWAS, genome‐wide association studies; HIF‐1α, the α subunit of hypoxia‐inducible factor‐1.

Carnitine concentration is altered in AD brains,^179^ suggesting that BBOX1 and TMLHE might play roles in AD progression. There is evidence that *L*‐carnitine and its acetylated derivative acetyl‐L‐carnitine (ALCAR) have therapeutic potential for neuroprotection.^180^ The accumulation of lipids in the extracellular space, a hallmark of AD,^181^ may reflect reduced *L*‐carnitine availability, possibly in turn due to reduced BBOX1 and TMLHE activity. A meta‐analysis at the mRNA level found that *BBOX1* was upregulated in AD across 12 datasets.^116^ It is possible that the brain enhances production of carnitine biosynthesis enzymes to compensate for their decreased activity.

#### 3.5.2 PHYH

Phytanoyl CoA hydroxylase plays a key role in the metabolism of phytanic acid, which is derived from the hydrophobic side chain of chlorophyll from the diet. After CoA‐esterification outside the peroxisome, phytanoyl CoA is transported into the peroxisome where it undergoes α‐oxidation due to the presence of a 3‐methyl‐group, which blocks the more normal β‐oxidation.^182^ PHYH catalyzes the second step in the α‐oxidation of phytanic acid in peroxisomes, in which phytanoyl‐CoA is converted to 2‐hydroxyphytanoyl‐CoA;^183^ the latter is subsequently converted to pristanoyl‐CoA, which can undergo β‐oxidization,^183^ producing dimethylnonanoyl‐CoA, which is finally exported as an acyl‐carnitine ester to the mitochondria, where further β‐oxidation occurs.^183^ The unprocessed form of PHYH contains a peroxisomal targeting sequence, which is cleaved on transport into peroxisomes.^183^

Peroxisomal function declines with age and peroxisomal dysfunction in AD has been extensively reviewed.^184^ Alterations in peroxisomal function are found in AD post mortem brain tissues^185^ and are proposed to contribute to neurodegenerative changes.^186^ Accumulation of very long chain fatty acids (VLCFAs), a symptom of peroxisomal dysfunction, is related to Aβ and tau toxicity,^184^ and may partially be caused by reduced peroxisomal α‐oxidation.^186^ Further studies are needed to explore the relationships among PHYH, VLCFAs, peroxisomal dysfunction, and AD.

The brain‐specific protein phytanoyl‐CoA‐hydroxylase associated protein 1 (PHYHIP), is reported to interact with PHYH via the *C*‐terminal region of the dual‐specificity tyrosine‐phosphorylated and regulated kinase 1A (DYRK1A), an interaction that may induce relocalization of nuclear DYRK1A to the cytosol.^187^ DYRK1A mRNA levels in the hippocampus were found to be significantly upregulated in patients with AD compared to pathological controls and DYRK1A could bridge Aβ production and tau phosphorylation.^188^ The potential role of PHYH in peroxisomal dysfunction or participation in the pathogenesis of AD via pathways, such as PHYH/PHYHIP/DYRK1A, merits further investigation.

### 3.6 2OGDD involved in collagen biosynthesis: Possible keys to repairing the ECM homeostasis in AD brains

Collagens are key components of the fibrillar and microfibrillar networks of the ECM and basement membranes.^189^ Various studies have shown that in the brain the ECM plays an important role in AD pathogenesis. Different types of collagens are upregulated in the AD brain; the functions of these in AD are not established.^30^ 2OGDD play key roles in collagen biosynthesis by catalyzing procollagen C‐3 and C‐4 prolyl hydroxylation (C‐P3Hs, C‐P4Hs) and lysyl C‐5 hydroxylation (PLODs)^177^ (Table 3).

The C‐P3H subfamily consists of leucine proline‐enriched proteoglycan (leprecan) 1 (LEPRE1) and leprecan‐like 1/2 (LEPREL1/2), also known as prolyl 3‐hydroxylase 1/2/3 (P3H1/2/3). P3H1 catalyzes formation of 3‐hydroxyproline in Pro‐Gly‐rich sequences in collagens (especially types IV and V); P3H2 catalyzes the post‐translational formation of 3‐hydroxyproline in ‐Gly‐Pro‐Hyp sequences where Hyp is 4‐hydroxyproline in collagens.^190^ P3H3 forms part of a complex consisting of PLOD1, P3H3, and P3H4 that catalyzes hydroxylation of collagen alpha chains. This modification is required for normal assembly and cross‐linking of collagen fibrils. The collagen prolyl‐4‐hydroxylase (C‐P4H) subfamily comprises prolyl 4‐hydroxylase subunit alpha‐1/2/3 (P4HA1/2/3), which catalyze/formation of 4‐hydroxyproline in –Xaa–Pro–Gly– sequences in collagens and other proteins. The pro‐collagen lysyl oxygenase domains (PLODs) are procollagen C‐5 lysyl hydroxylases. PLOD1 and PLOD2 form hydroxylate lysine residues in –X–Lys–Gly– sequences in collagens.^191^, ^192^, ^193^ These hydroxylysines function as attachment sites for carbohydrates that are essential for the collagen stability/intermolecular cross‐linking. PLOD3 is a multifunctional enzyme that catalyzes essential post‐translational modifications of Lys residues in procollagen.^194^, ^195^, ^196^, ^197^, ^198^

Meta‐analysis of four GWAS showed *P4HA1* is in the top 5% of genes associated with AD.^74^ *P4HA2* is a HIF‐target gene in astrocytes.^199^ A meta‐analysis of four GWAS shows *PLOD1* is in the top 5% of genes associated with AD.^74^ There is a significant increase in *PLOD1* in whole blood from AD fast progressors compared to slow progressors^24^ (fast progressors: early AD patients with changes in Clinical Dementia Rating‐Sum of Boxes [CDR‐SB] scores ≥2 points/year; slow‐progressors are those with CDR‐SB changes of <2 points/year^24^). Thus, PLOD1 is a potential prognostic blood biomarker in early AD.^24^ Another meta‐analysis of AD at the mRNA level showed *PLOD3* was significantly upregulated in AD across 12 datasets.^116^ *PLOD1* and *PLOD2* have been shown to be HIF targets,^200^ including in astrocytes.^199^ Overall, there is a good case for further investigations of the roles of the PLODs in AD.

It is possible that the PLODs and C‐P4Hs have roles relating to hypoxia in AD. P4HA1 is reported to enhance the stability of HIF‐1α protein in breast cancer cells, possibly by reducing 2‐OG levels and/or increasing succinate production.^201^ HIF‐1α has been shown to promote ECM remodeling under hypoxic conditions, likely in part by inducing P4HA1, P4HA2, and PLOD2 expression in fibroblasts.^202^ It is possible that a positive feedback mechanism involving P4HA1/2 (and the PLODs) and HIF modulates collagen biosynthesis and hence ECM biochemistry. A relationship between P3H1/2/3 and AD remains to be defined, though their genes are reported to be related to AD.^203^

### 3.7 2OGDD acting on nucleobases and nucleic acids

Nucleobases/nucleic acids are a major class of oligomeric 2OGDD substrates.^177^ Human 2OGDD acting on nucleic acids, are alkylation repair enzyme homologs (ALKBH 1‐8), the TETs, and the fat mass and obesity‐associated (FTO) protein (Table 4). 2OGDD acting on nucleobases are widely distributed in aerobic biology, including in bacteria, where AlkB has a validated role in DNA and, likely, RNA damage repair due to *N*‐alkylation; AlkB catalyzes demethylation via initial hydroxylation followed by fragmentation to give an aldehyde (formaldehyde in the case of *N*‐methyl demethylation).^204^ The functions of eukaryotic 2OGDD acting on nucleic acids are more complex, though they likely have roles in damage repair and epigenetic regulation, in the latter case acting on DNA, RNA, and, maybe, proteins.

**TABLE 4:** Human 2OGDD acting on nucleobases and nucleic acids, their expression levels, and potential roles in AD

2OGDD name	Abbv.	Altered expression in AD	Cell lines/models/samples	Known and potential functions relating to AD	Enzymatic activity	Refs.
Ten‐eleven translocation 1	TET1	Upregulation in Hippocampus and Parahip pocampal gyrus	Preclinical AD and late‐stage AD human subjects	Catalyzing formation of 5hmC, 5‐formyl C and 5‐carboxy C Affecting binding of transcription factors	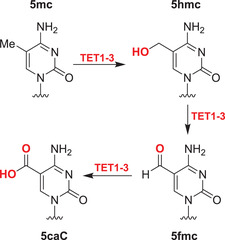	^245^
		Downregulation in the hippocampus and prefrontal cortex	Postmortem human AD hippocampus; 3×Tg mice			^246^
Ten‐eleven translocation 2	TET2	Upregulation in plaque‐associated microglia; downregulation in the hippocampus and prefrontal cortex	Human post‐mortem brain tissue specimens; 2×Tg‐AD mice; 5xFAD mice; 3×Tg mice	Catalyzing formation of 5hmC, 5‐formyl C and 5‐carboxy C Regulating neuroinflammation and cognitive function		^246^, ^250^, ^251^
Ten‐eleven translocation 3	TET3	Downregulation in the hippocampus hippocampus and prefrontal cortex	APP/PS1 mice; 3×Tg mice; Postmortem human AD hippocampus	Catalyzing formation of 5hmC, 5‐fC and 5‐caC. Regulation in the hippocampal neurogenesis, neuronal differentiation of neural stem cells and cognitive function		^246^, ^247^
AlkB homolog 1	ALKBH1				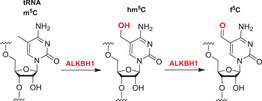	^177^
AlkB homolog 2	ALKBH2					
AlkB homolog 3	ALKBH3			Induced by AICD, involved in DNA damage repair, and APP signaling	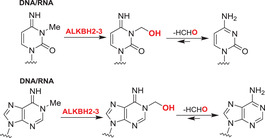	^223^, ^224^, ^225^
AlkB homolog 4	ALKBH4				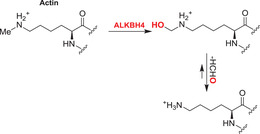	^177^
AlkB homolog 5	ALKBH5	Downregulation in entorhinal cortex	incipient AD cases	Hydroxylating mRNA m6A. Changing synaptic function	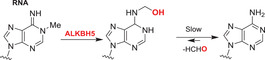	^230^
Fat‐, mass‐, and obesity‐ associated protein	FTO	Controversial	3xTg‐AD mice APP/PS1 mice	Hydroxylating mRNA m6A. Changing synaptic function; activating phosphorylation of tau in an mTOR‐dependent manner	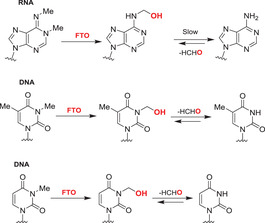	^237^, ^238^, ^239^, ^240^
AlkB homolog 6	ALKBH6	Downregulation in entorhinal cortex and the whole brain (minus cerebellum)	incipient AD cases Tg2576 mice			^230^, ^231^
AlkB homolog 7	ALKBH7					
AlkB homolog 8	ALKBH8				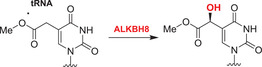	

*Note*: Table is not comprehensive both with respect to the reactions and roles of the 2OGDD.

Abbreviations: AD, Alzheimer's disease; AICD, the intracellular domain of amyloid precursor protein (APP); HIFs, hypoxia‐inducible factors; 5hmc (hm^5^C), 5‐hydroxymethylcytosine; m^5^C, 5‐methylcytosine; f^5^C, 5‐formylcytosine; m6A, *N*6‐methyladenine.

#### 3.7.1 AlkB homologues

Although further work is required, like bacterial AlkB, there is evidence that ALKBH2 and ALKBH3 repair alkylated DNA and RNA containing 1‐methyladenine (m1A) and 3‐methylcytosine (m3C) by oxidative demethylation.^205^, ^206^, ^207^, ^208^ ALKBH1 is reported to be an *N*‐methyl mitochondrial nucleic acid demethylase^209^ and to have apyrimidinic/apurinic lyase activity;^210^, ^211^, ^212^ ALKBH1 is thought to act predominantly on tRNAs and to mediate oxidative demethylation in a context and subcellular compartment‐dependent manner.^213^, ^214^ ALKBH4 is reported to be involved in regulating actomyosin processes, possibly by mediating demethylation of monomethylated (K84me1) actin.^215^ ALKBH7 is involved in programmed necrosis induced by DNA damage mediated by cytotoxic alkylating agents.^216^, ^217^, ^218^ ALKBH8,^219^, ^220^, ^221^, ^222^ which participates in tRNA maturation, contains both an *S*‐adenosylmethionine‐dependent methyltransferase domain, which is required for the generation of 5‐methoxycarbonylmethyluridine (mcm5U) by methylation of 5‐carboxymethyluridine (cm5U), and a dioxygenase domain, which generates (*S*)‐5‐methoxycarbonylhydroxymethyluridine (mchm5U) by hydroxylation of mcm5U.^220^, ^221^

Reverse transcription polymerase chain reaction (RT‐PCR) analyses have shown the intracellular domain of APP (AICD) strongly enhances the expression of *ALKBH3*.^223^ AICD, which is increased in AD brains,^224^, ^225^ seems to act as an indirect regulator of DNA damage response and repair functions.^225^ Investigations on the potential role of ALKBH3 in APP signaling are of interest.

The primary function of ALKBH5 appears to be demethylation of *N*6‐methyladenine (m6A) in mRNA.^226^, ^227^, ^228^, ^229^ Laser capture microdissection (LCM)‐based expression profiling of neurons has shown that that the *ALKBH5* and *ALKBH6* genes are significantly downregulated in incipient AD cases,^230^ indicating they may be involved in AD pathogenesis. The function and substrates of ALKBH6 remain to be established. Serial analysis of gene expression (SAGE) studies indicate *ALKBH6* expression is downregulated in the Tg2576 mouse model of AD.^231^ The combined results of these studies imply *ALKBH6* expression is related to AD; the potential association of ALKBH6 with AD thus merits further evaluation. *ALKBH5* has been confirmed as a HIF‐1α target gene, that is, is induced by hypoxia, which is characteristic of AD development.^232^ Future studies can focus on how, if at all, hypoxia‐regulated ALKBH5 catalyzed mRNA m6A oxidation relates to AD.

#### 3.7.2 FTO

Like ALKBH5, the primary function of FTO appears to be oxidation of m6A in mRNA, although FTO, the overexpression of which is closely linked to obesity and diabetes,^233^, ^234^, ^235^ also oxidizes the methyl groups of 3‐methylthymine (m3T) and 3‐methyluracil (m3U) in ssDNA.^236^ Links between FTO and AD have been reviewed,^237^ focusing mainly on FTO/mTOR‐induced tau phosphorylation in AD. It has been shown that carriers of the *FTO* AA‐genotype have an increased risk of AD, possibly through the interaction with apolipoprotein E ε4.^238^ It is proposed that the *FTO* gene is regulated in AD via a leptin‐regulated pathway activated by factors such as a high midlife body mass index (BMI), which has an independent and direct effect on the central nervous system (CNS).^238^ The mechanism by which the *FTO* gene influences the risk of AD requires further investigation.

Two recent papers reports come to opposing conclusions concerning protein expression of FTO in AD brains in model studies. Elevated levels of FTO were found in the brain of 3xTg‐AD mice, leading to inhibition of tuberous sclerosis complex 1 (TSC1) and an elevation in mTOR signaling, which is critical in AD pathology.^239^ On the other hand, FTO was decreased in APP/PS1 mice brains, consistent with the proposal increased m6A methylation that promotes the development of AD.^240^ Further work is thus required both to optimize models and to define expression level of FTO in human AD brains.

#### 3.7.3 TET proteins

The TET subfamily, comprising TET1‐3, catalyzes sequential 5mC oxidation to generate 5hmC, 5fmC, and then 5caC in both DNA and RNA^15^, ^241^ (Table 4). The roles of TETs in neurophysiology and brain function have been summarized.^242^ The TETs may play roles in the regulation of neurogenesis and cognitive functions by mediating 5mC oxidation in important neuronal regulatory genes.^242^ Thus, as with the JmjC KDMs, modulation of TET‐mediated epigenetic changes in AD progress might improve neuron/synapse function and cognitive process.

The results of genome‐wide profiles of both 5mC and 5hmC in the human frontal cortex tissues from late‐onset Chinese AD patients and cognitively normal controls, suggest a role for 5hmC in AD pathogenesis.^243^ Interestingly, HIF‐2α and HIF‐1α were found to be enriched in differentially hydroxymethylated regions (DhMRs) of the brain, indicating that altered 5hmC status may regulate expression by altering the binding of HIF to its target genes, in a manner contributing to pathogenesis of AD.^243^ This proposal is consistent with structural and biochemical studies on 5hmC DNA duplexes implying 5hmC can induce context dependent regulation of transcription factor (including HIF) binding.^244^ The work indicating that the TETs and HIF‐α may cooperate in regulation of expression in AD is important as it provides a pathophysiologically relevant link between HIF‐α and 2OGDD in addition to the already validated PHD/FIH connection (see Section 2 above).

Levels of TET1, 5mC, and 5hmC are reported to be increased in AD brains, while levels of 5‐formylcytosine (5fmC) and 5‐carboxylcytosine (5caC) are decreased.^245^ By contrast, in human and 3×TG mice AD brains, decreased levels of TETs are responsible for selective loss of 5hmC^246^; thus, 5hmC‐mediated epigenetic dysregulation may contribute to progression of neurodegeneration in AD.^246^

TET3 is reported to be downregulated through activation of the IL‐6/JAK2/STAT3 pathway in the hippocampus of APP/PS1 mice.^247^ Dysfunction of TET3‐mediated regulation of 5hmC levels leads to abnormal neuronal function‐related gene expression, contributing to cognitive deficits and inhibited hippocampal neurogenesis in APP/PS1 mice.^247^ The loss of TET2 resulted in the upregulation of several inflammatory mediators, including interleukin (IL)‐6,^248^ which may account for the high levels of IL‐6 detected in the hippocampus of APP/PS1 mouse. It seems that TET2 may work with TET3 to regulate AD‐related gene expression via the IL‐6/JAK2/STAT3 pathway.

Chronic inflammation and Aβ accumulation are established features of AD.^249^ Recent studies have demonstrated an association between TET2 and neuroinflammation in AD.^250^ TET2 is highly upregulated in Aβ plaque‐associated microglia in both AD transgenic mice (5×FAD) and human AD tissues.^250^ TET2 acts as a regulator of the microglial proinflammatory response by regulating genes related to the type I interferon response;^250^ further studies are needed to evaluate the impact of microglial TET2 in AD. TET2 is of interest as a potential target for regulation of the exacerbated neuroinflammatory response in AD.^250^ Significant upregulation of PHD3 was also observed in Aβ plaque‐associated microglia.^77^ The association between PHD3 and TET2 in PAM merits further exploration.

By contrast, a significant decrease in 5hmC and TET2 levels was observed in the hippocampus of aged 2×Tg‐AD mice;^251^ it was suggested that reduced TET2 triggered neuroinflammation in the brains of early‐stage AD patients, resulting in exacerbation of AD progression.^251^ TET2 is reported to be required to resolve inflammation by recruiting histone deacetylase 2 (HDAC2) to specifically repress IL‐6,^248^ consistent with the proposal that TET2 may contribute to chronic inflammation in AD. The interaction of TET2 and histone deacetylase 1 (HDAC1) is involved in regulation of 5hmC levels and expression of the downstream genes associated with AD pathology and cognitive deficits.^251^ Hence, the role of the TET2/HDAC2/IL‐6 axis in AD deserves further studies. Targeting the TET2/HDAC1/2‐regulalion of expression may be a novel therapeutic approach to AD treatment.

### 3.8 Other 2OGDD

#### 3.8.1 EGFH

The human epidermal growth factor aspartate/asparaginyl hydroxylase (EGFH) subfamily contains three members, that is ASPH, aspartate‐β‐hydroxylase domain‐containing protein 1 (ASPHD1), and aspartate‐β‐hydroxylase domain‐containing protein 2 (ASPHD2). ASPH catalyzes the C‐3 hydroxylation of aspartyl and asparaginyl residues in the epidermal growth factor‐like domains of proteins such as Notch and its homologs,^252^ while the enzymatic activities of ASPHD1/2 are uncharacterized (Table 5). Recent work has shown the epidermal growth factor‐like domain (EGFD) substrates of ASPH to have a non‐canonical disulfide pattern and that the kinetics of ASPH are consistent with a role in hypoxia sensing.^253^, ^254^ ASPH also has an unusual Fe(II) binding geometry in that only two proteins residues that chelate the active site metal.^255^ ASPH hydroxylated proteins (e.g., clotting factors) are present in blood; analysis of the hydroxylation status of these is of interest from an AD biomarker perspective.

**TABLE 5:** Summary of other human 2OGDD, their expression levels and potential roles in AD

2OGDD name	Abbreviation	Altered expression in AD	Cell lines/models/samples	Known and potential functions relating to AD	Enzymatic activity	Refs.
Epidermal growth factor aspartate/asparaginyl hydroxylase	ASPH, EGFH			EGF domain hydroxylation. Hypoxially regulated and possible interactions with HIF‐1α/involved in hypoxia‐inducible gene regulation and neuronal motility	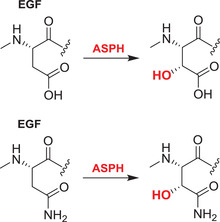	^256^
Aspartate‐β‐hydroxylase domain‐containing protein 1	ASPHD1					
Aspartate‐β‐hydroxylase domain‐containing protein 1	ASPHD2					
2‐Oxoglutarate (2OG) and Fe(II)‐dependent oxygenase domain containing protein 1	OGFOD1			Positively regulating eIF2α phosphorylation, contributing to the pathogenesis of AD	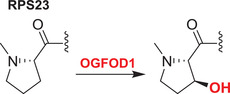	^258^, ^259^, ^260^, ^261^
2‐Oxoglutarate (2OG) and Fe(II)‐dependent oxygenase domain containing protein 2	OGFOD2					
2‐Oxoglutarate (2OG) and Fe(II)‐dependent oxygenase domain containing protein 3	OGFOD3 (C17orf101)					
Prolyl‐4‐hydroxylase transmembrane domain	P4HTM	AD‐related gene	GWAS Meta‐analysis	Regulating expression of HIF‐α target genes, such as EPO	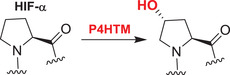	^74^, ^264^
Phytanoyl‐CoA dioxygenase domain‐containing protein 1	PHYHD1	Upregulation in the entorhinal, temporal and frontal cortices	APP^NL‐G‐F/NL‐G‐F^ mice Human postmortem brain tissue specimens	Participating in cortical Aβ amyloidosis formation, progression of neurofibrillary tangles and neuroinflammation		^265^, ^266^, ^267^, ^268^, ^280^

*Note*: Table is not comprehensive, both with respect to the reactions and roles of 2OGDD.

Abbreviations: Aβ, amyloid beta; AD, Alzheimer's disease; EGF, epidermal growth factor; EPO, erythropoietin; eIF2α, eukaryotic initiation factor 2 α‐subunit; GWAS, genome‐wide association studies; HIF‐1α, the α subunit of hypoxia‐inducible factor‐1; RPS23, ribosomal protein S23.

Lawton et al. report evidence for cross‐talk among ASPH, HIF‐1α, and Notch in the regulation of neuronal motility, suggesting a possible role for ASPH in AD.^256^ Specifically, mRNA levels of ASPH and HIF‐1α were reported to be upregulated through inhibition of GSK‐3β; expression of hypoxia‐inducible genes was promoted by insulin and insulin‐like growth factor, type 1 (IGF‐1),^256^ either directly or via the ASPH/Notch/Jagged/HES‐1 pathway. Impaired insulin signaling is characteristic of AD and abnormal insulin signaling may activate the ASPH/Notch/HES‐1 axis to regulate the expression of hypoxia‐inducible genes.

#### 3.8.2 OGFODs

The 2OG and Fe(II)‐dependent oxygenase domain‐containing protein (OGFOD) subfamily contains three members, OGFOD1–3. OGFOD1 has been shown to catalyze hydroxylation of Pro‐62 of the small ribosomal subunit protein RPS23 and may have a role in the regulation of translation, though this has not been demonstrated in humans^257^ (Table 5). Few studies have focused on OGFOD2 and OGFOD3 (C17orf101) and their functions are unknown. It is reported that OGFOD1 can interact with both eukaryotic initiation factor 2 α‐subunit (eIF2α) and the eIF2α kinase heme‐regulated inhibitor (HRI), further positively regulating eIF2α phosphorylation.^258^ Elevated eIF2α phosphorylation has been observed in the brains of AD patients and model mice^259^ and is reported to contribute to pathogenesis of Aβ overproduction^260^ and neuronal degeneration.^261^ eIF2α is phosphorylated by four kinases: double‐stranded RNA‐dependent protein kinase (PKR), PKR‐like endoplasmic reticulum kinase (PERK), heme‐regulated eIF2α kinase (HRI), and amino acid‐regulated eIF2α kinase (GCN2). Suppression of PERK and GCN2 was found to alleviate AD‐related plasticity and memory deficits by preventing hyperphosphorylation of eIF2α.^259^ Hence, blocking the OGFOD1/HRI/eIF2α axis may produce the similar effects. Further studies are needed to investigate OGFOD1 as a potential therapeutic target in AD.

#### 3.8.3 P4HTM

Transmembrane prolyl 4‐hydroxylase (P4HTM) is a member of the prolyl 4‐hydroxylase (P4H) structural 2OGDD family and its sequence more closely resembles those of the C‐P4Hs than the PHDs.^262^ *P4HTM* is highly expressed in the brain and, like the PHDs, there is evidence that it participates in O_2_‐dependent regulation of HIF‐α isoforms.^263^ P4HTM is reported to catalyze the post‐translational formation of 4‐hydroxyproline in HIF‐α (Table 5); consistent with this P4HTM is involved in the regulation of EPO production in the kidney.^264^ Whether P4HTM regulates EPO levels in the AD brain requires further investigation. Notably, a meta‐analysis of four GWAS showed that *P4HTM* is one of the top 5% of genes associated with AD.^74^

#### 3.8.4 PHYHD1

There are three known isoforms of PHYHD1, of which only PHYHD1A has been shown to be a functional 2OG oxygenase.^265^ Although its substrate(s)/and role in AD are unclear, clear links between PHYHD1 and AD are reported (Table 5). Notably, PHYHD1 is reported to make a direct interaction with Aβ_42_.^268^ Human microarray data analysis shows that expression of *PHYHD1* is substantially increased in human AD cortices, especially in the temporal cortices.^267^ Increased *phyhd1* expression was also observed in APP^NL‐G‐F/NL‐G‐F^ mice, which are characterized by Aβ amyloidosis and neuroinflammation.^267^ PHYHD1 has been categorized as an inflammatory response and AD‐related protein through biological function analysis using pathway analysis, a link which may relate to the age‐dependent increase in *phyhd1* expression in APP^NL‐G‐F/NL‐G‐F^ mice.^267^

Increased expression of *PHYHD1* and unconventional myosin‐Vc (*MYO5c*) are associated with Braak NFT stage progression in human AD brains.^268^ Protein–protein interaction network analysis indicates the interaction between PHYHD1 and MYO5C is mediated by protection of telomeres protein 1 (POT1).^268^ Compared to control individuals, marked changes in *PHYHD1* and *MYO5c* expression levels were found in patients with LOAD, indicating that they may be involved in the development of AD.^268^

Overall, PHYHD1 is a 2OGDD of particular interest to AD. There is good evidence linking *PHYHD1*, which is a highly expressed gene in human AD brains to cortical Aβ amyloidosis, NFT progression, and the neuroinflammatory response, possibly via the PHYHD1/POT1/MYO5C axis. Stimulation of naïve T cells resulted in upregulation of *PHYHD1* after stimulation leading to differentiation into effector T cells.^269^ T cell infiltration in the brain of AD patients has been reported.^270^, ^271^, ^272^ Whether upregulation of *PHYHD1* is related to T cell infiltration in AD brains merits further exploration.

## CONFLICTS OF INTEREST

The authors declare no competing financial interests. Author disclosures are available in the supporting information.

## Supporting information

SUPPORTING INFORMATION

SUPPORTING INFORMATION

SUPPORTING INFORMATION

SUPPORTING INFORMATION
