# Supplementary material for: Exploring links between 2‐oxoglutarate‐dependent oxygenases and Alzheimer's disease
Source: Alzheimers Dement. 2022 Jul 19;18(12):2637–68. doi: 10.1002/alz.12733 (PMC10083964; doi:10.1002/alz.12733)
Supplement: Supplementary file 2 — SUPPORTING INFORMATION [file ALZ-18-2637-s001.pdf]

Supplementary Table 1. Domain organization and substrates of 2OGDD human protein hydroxylases.				
**Gene ID**	**2OGDD**	**Domain architecture**	**Substrates / Proposed Substrates***	**Ref.**
444	ASPH	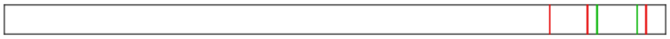	Selected epidermal growth factor-like (EGF) domains of multiple of proteins	1
112399	EGLN1 (PHD2)	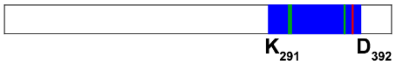	HIF-α isoforms / potentially non HIF-α substrates	2,3
54583	EGLN2 (PHD1)	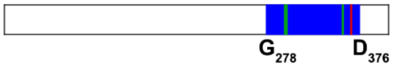	HIF-α isoforms / potentially non HIF-α substrates, e.g. Cep192, IKBKB	2-6
112398	EGLN3 (PHD3)	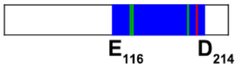	HIF-α isoforms, / potentially non HIF-α substrates, e.g. ADRB2, TELO2, PKM2	2,3,7,8
55662	HIF1AN (FIH)	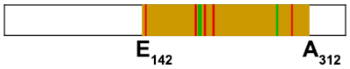	HIF-α isoforms, ankyrin repeat domains containing proteins (e.g. ASB4, NOTCH1, MYPT1), other substrates	9-11
65094	JMJD4	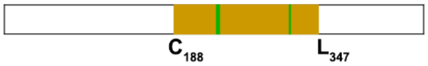	eRF1	12
23210	JMJD5 (KDM8)	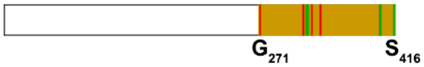	RPS6, RCCD1, methylated arginines of histone H2, H3 and H4 (all to be validated in cells)	13-15
8681	JMJD6	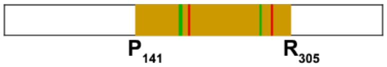	U2AF2 / U2AF65 (and other splicing regulatory proteins), potentially H3R2me, H4R3me, arginine methylated proteins (e.g. ESR1)	16-19
337123	JMJD7	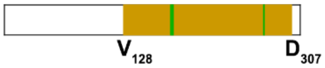	TRAFAC, DRG1, potentially methylated arginines of histone H2, H3 and H4 (further validation required)	15,20
64175	LEPRE1 (P3H1)	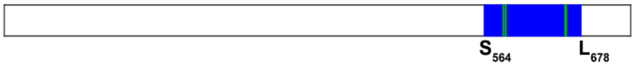	procollagens (especially types IV and V)	21
55214	LEPREL1 (P3H2)	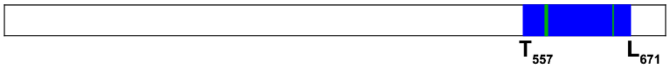	procollagens (especially types IV)	22
10536	LEPREL2 (P3H3)	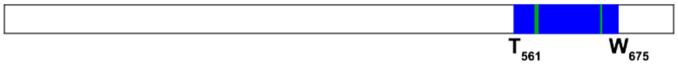	Unknown	
84864	MINA53 (RIOX2)	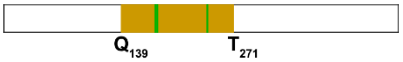	RPL27A, H3K9me3 (further validation required)	23,24

79697	NO66 (RIOX1)	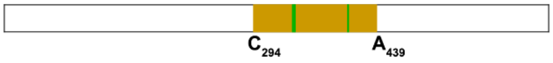	RPL8, H3K4me1, H3K4me3, H3K36me2 (further validation required)	23,25
55239	OGFOD1	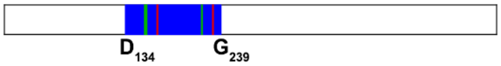	RPS23	26,27
5033	P4HA1	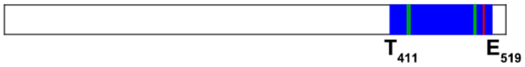	Procollagens and proteins containing collagenous domains, e.g. adiponectin	
8979	P4HA2	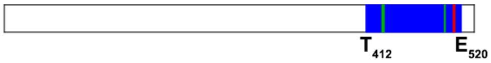	Procollagens and proteins containing collagenous domains, e.g. adiponectin	
283208	P4HA3	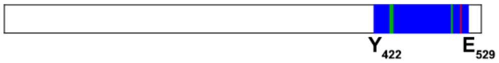	Procollagens and proteins containing collagenous domains, e.g. adiponectin	
54681	P4HTM	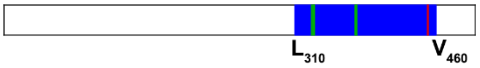	HIF-1α (further validation required)	28
5351	PLOD1	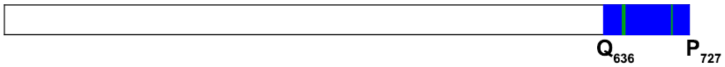	Procollagens and proteins containing collagenous domains, e.g. adiponectin	29,30
5352	PLOD2	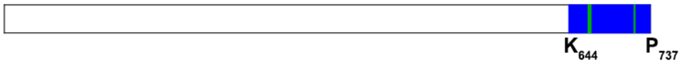	Procollagens and proteins containing collagenous domains, e.g. adiponectin	
8985	PLOD3	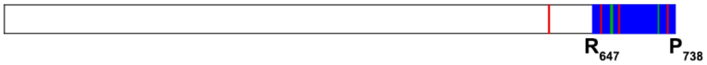	Procollagens and proteins containing collagenous domains, e.g. adiponectin, mannan-binding lectin	31-35

: Catalytic domain : JmjC domain : Fe^{2+} binding sites : 2OG binding sites

[*] Lists of (potential) substrates are not exhaustive / some potential substrates need to be validated.

1. Dinchuk JE, Focht RJ, Kelley JA, et al. Absence of post-translational aspartyl beta-hydroxylation of epidermal growth factor domains in mice leads to developmental defects and an increased incidence of intestinal neoplasia. *J Biol Chem.* 2002;277(15):12970-12977.
2. Epstein AC, Gleadle JM, McNeill LA, et al. C. elegans EGL-9 and mammalian homologs define a family of dioxygenases that regulate HIF by prolyl hydroxylation. *Cell.* 2001;107(1):43-54.
3. Cockman ME, Lippl K, Tian Y-M, et al. Lack of activity of recombinant HIF prolyl hydroxylases (PHDs) on reported non-HIF substrates. *Elife.* 2019;8.
4. McNeill LA, Hewitson KS, Gleadle JM, et al. The use of dioxygen by HIF prolyl hydroxylase (PHD1). *Bioorg Med Chem Lett.* 2002;12(12):1547-1550.
5. Cummins EP, Berra E, Comerford KM, et al. Prolyl hydroxylase-1 negatively regulates IkappaB kinase-beta, giving insight into hypoxia-induced NFkappaB activity. *Proc Natl Acad Sci USA.* 2006;103(48):18154-18159.
6. Moser SC, Bensaddek D, Ortmann B, et al. PHD1 links cell-cycle progression to oxygen sensing through hydroxylation of the centrosomal protein Cep192. *Dev Cell.* 2013;26(4):381-392.
7. Luo W, Hu H, Chang R, et al. Pyruvate kinase M2 is a PHD3-stimulated coactivator for hypoxia-inducible factor 1. *Cell.* 2011;145(5):732-744.
8. Xie L, Xiao K, Whalen EJ, et al. Oxygen-regulated beta(2)-adrenergic receptor hydroxylation by EGLN3 and ubiquitylation by pVHL. *Sci Signal.* 2009;2(78):ra33.
9. Cockman ME, Lancaster DE, Stolze IP, et al. Posttranslational hydroxylation of ankyrin repeats in IkappaB proteins by the hypoxia-inducible factor (HIF) asparaginyl hydroxylase, factor inhibiting HIF (FIH). *Proc Natl Acad Sci USA.* 2006;103(40):14767-14772.
10. Webb JD, Murányi A, Pugh CW, Ratcliffe PJ, Coleman ML. MYPT1, the targeting subunit of smooth-muscle myosin phosphatase, is a substrate for the asparaginyl hydroxylase factor inhibiting hypoxia-inducible factor (FIH). *Biochem J.* 2009;420(2):327-333.
11. Lando D, Peet DJ, Gorman JJ, Whelan DA, Whitelaw ML, Bruick RK. FIH-1 is an asparaginyl hydroxylase enzyme that regulates the transcriptional activity of hypoxia-inducible factor. *Genes Dev.* 2002;16(12):1466-1471.
12. Feng T, Yamamoto A, Wilkins SE, et al. Optimal translational termination requires C4 lysyl hydroxylation of eRF1. *Mol Cell.* 2014;53(4):645-654.
13. Wilkins SE, Islam MS, Gannon JM, et al. JMJD5 is a human arginyl C-3 hydroxylase. *Nat Commun.* 2018;9(1):1180.
14. Shen J, Xiang X, Chen L, et al. JMJD5 cleaves monomethylated histone H3 N-tail under DNA damaging stress. *EMBO Rep.* 2017;18(12):2131-2143.
15. Liu H, Wang C, Lee S, et al. Clipping of arginine-methylated histone tails by JMJD5 and JMJD7. *Proc Natl Acad Sci USA.* 2017;114(37):E7717-E7726.
16. Poulard C, Rambaud J, Hussein N, Corbo L, Le Romancer M. JMJD6 regulates ERα methylation on arginine. *PLoS ONE.* 2014;9(2):e87982.
17. Chang B, Chen Y, Zhao Y, Bruick RK. JMJD6 is a histone arginine demethylase. *Science (New York, NY).* 2007;318(5849):444-447.
18. Webby CJ, Wolf A, Gromak N, et al. Jmjd6 catalyses lysyl-hydroxylation of U2AF65, a protein associated with RNA splicing. *Science (New York, NY).* 2009;325(5936):90-93.
19. Liu W, Ma Q, Wong K, et al. Brd4 and JMJD6-associated anti-pause enhancers in regulation of transcriptional pause release. *Cell.* 2013;155(7):1581-1595.
20. Markolovic S, Zhuang Q, Wilkins SE, et al. The Jumonji-C oxygenase JMJD7 catalyzes (3S)-lysyl hydroxylation of TRAFAC GTPases. *Nature chemical biology.* 2018;14(7):688-695.
21. Kaul SC, Sugihara T, Yoshida A, Nomura H, Wadhwa R. Gros1, a potential growth suppressor on chromosome 1: its identity to basement membrane-associated proteoglycan, leprecan. *Oncogene.* 2000;19(32):3576-3583.
22. Tiainen P, Pasanen A, Sormunen R, Myllyharju J. Characterization of recombinant human prolyl 3-hydroxylase isoenzyme 2,

an enzyme modifying the basement membrane collagen IV. *J Biol Chem.* 2008;283(28):19432-19439.

23. Ge W, Wolf A, Feng T, et al. Oxygenase-catalyzed ribosome hydroxylation occurs in prokaryotes and humans. *Nature chemical biology.* 2012;8(12):960-962.

24. Lu Y, Chang Q, Zhang Y, et al. Lung cancer-associated JmjC domain protein mdig suppresses formation of tri-methyl lysine 9 of histone H3. *Cell Cycle.* 2009;8(13):2101-2109.

25. Sinha KM, Yasuda H, Coombes MM, Dent SYR, de Crombrugghe B. Regulation of the osteoblast-specific transcription factor Osterix by NO66, a Jumonji family histone demethylase. *EMBO J.* 2010;29(1):68-79.

26. Singleton RS, Liu-Yi P, Formenti F, et al. OGFOD1 catalyzes prolyl hydroxylation of RPS23 and is involved in translation control and stress granule formation. *Proc Natl Acad Sci USA.* 2014;111(11):4031-4036.

27. Loenarz C, Sekirnik R, Thalhammer A, et al. Hydroxylation of the eukaryotic ribosomal decoding center affects translational accuracy. *Proc Natl Acad Sci USA.* 2014;111(11):4019-4024.

28. Koivunen P, Tiainen P, Hyvärinen J, et al. An endoplasmic reticulum transmembrane prolyl 4-hydroxylase is induced by hypoxia and acts on hypoxia-inducible factor alpha. *J Biol Chem.* 2007;282(42):30544-30552.

29. Pirskanen A, Kaimio AM, Myllylä R, Kivirikko KI. Site-directed mutagenesis of human lysyl hydroxylase expressed in insect cells. Identification of histidine residues and an aspartic acid residue critical for catalytic activity. *J Biol Chem.* 1996;271(16):9398-9402.

30. Yeowell HN, Allen JD, Walker LC, Overstreet MA, Murad S, Thai SF. Deletion of cysteine 369 in lysyl hydroxylase 1 eliminates enzyme activity and causes Ehlers-Danlos syndrome type VI. *Matrix Biol.* 2000;19(1):37-46.

31. Wang C, Luosujärvi H, Heikkinen J, Risteli M, Uitto L, Myllylä R. The third activity for lysyl hydroxylase 3: galactosylation of hydroxylysyl residues in collagens in vitro. *Matrix Biol.* 2002;21(7):559-566.

32. Wang C, Kovanen V, Raudasoja P, Eskelinen S, Pospiech H, Myllylä R. The glycosyltransferase activities of lysyl hydroxylase 3 (LH3) in the extracellular space are important for cell growth and viability. *J Cell Mol Med.* 2009;13(3):508-521.

33. Rautavuoma K, Takaluoma K, Passoja K, et al. Characterization of three fragments that constitute the monomers of the human lysyl hydroxylase isoenzymes 1-3. The 30-kDa N-terminal fragment is not required for lysyl hydroxylase activity. *J Biol Chem.* 2002;277(25):23084-23091.

34. Risteli M, Ruotsalainen H, Bergmann U, Venkatraman Girija U, Wallis R, Myllylä R. Lysyl hydroxylase 3 modifies lysine residues to facilitate oligomerization of mannan-binding lectin. *PLoS ONE.* 2014;9(11):e113498.

35. Ruotsalainen H, Risteli M, Wang C, et al. The activities of lysyl hydroxylase 3 (LH3) regulate the amount and oligomerization status of adiponectin. *PLoS ONE.* 2012;7(11):e50045.
