# Supplementary material for: Exploring links between 2‐oxoglutarate‐dependent oxygenases and Alzheimer's disease
Source: Alzheimers Dement. 2022 Jul 19;18(12):2637–68. doi: 10.1002/alz.12733 (PMC10083964; doi:10.1002/alz.12733)
Supplement: Supplementary file 3 — SUPPORTING INFORMATION [file ALZ-18-2637-s002.pdf]

Supplementary Table 2. Domain organization and substrates of N^{ε}-methyl lysine histone demethylases in 2OGDDs.				
**Gene ID**	**2OGDD**	**Domain architecture***	**Substrates / Proposed Substrates****	**Ref.**
22992	FBXL11 (KDM2A)	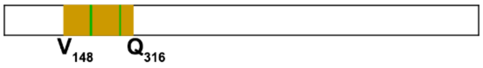	H3K36me1/me2, p65 subunit of NF-κB	1,2
84678	FBXL10 (KDM2B)	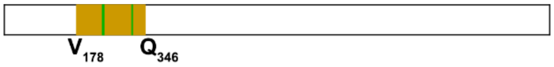	H3K36me1/me2, H3K4me3	2,3
55818	JMJD1A (KDM3A)	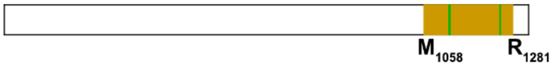	H3K9me1/me2	4
51780	JMJD1B (KDM3B)	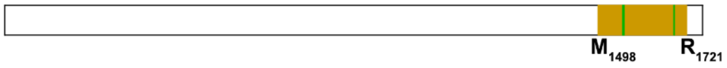	H3K9me1/me2	4
9682	JMJD2A (KDM4A)	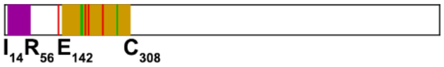	H3K9me3, H3K36me3	5,6
23030	JMJD2B (KDM4B)	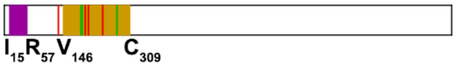	H3K9me3	5
23081	JMJD2C (KDM4C)	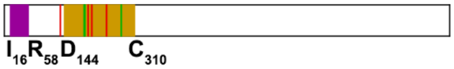	H3K9me3, H3K36me3	5
55693	JMJD2D (KDM4D)	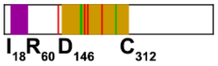	H3K9me2/me3	5
390245	JMJD2E (KDM4E)	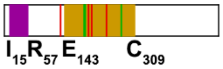	H3K9me2/me3	7
5927	JARID1A (KDM5A)	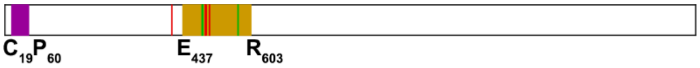	H3K4me2/me3	8
10765	JARID1B (KDM5B)	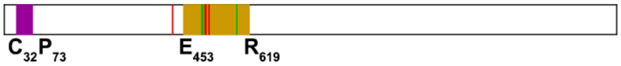	H3K4me1/me2/me3	9
8242	JARID1C (KDM5C)	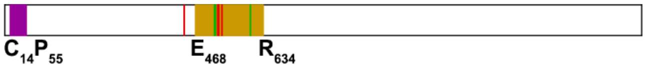	H3K4me2/me3	10-12
8284	JARID1D (KDM5D)	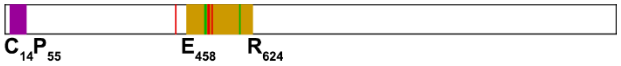	H3K4me2/me3	13
7403	UTX (KDM6A)	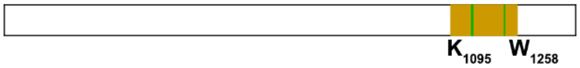	H3K27me2/me3	14-16
23135	JMJD3 (KDM6B)	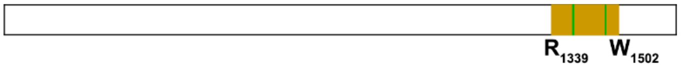	H3K27me2/me3	17,18
7404	UTY (KDM6C)	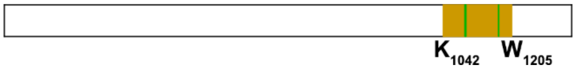	H3K27me3	19
80853	JHDM1D (KDM7A, KIA1718)	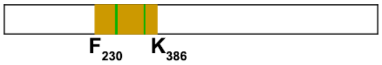	H3K9me2, H3K27me2, H4K20me1	20-22
23133	PHF8 (KDM7B)	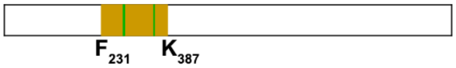	H3K9me1/me2, H3K27me2, H4K20me1	23-25
5253	PHF2	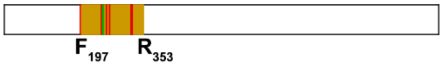	H3K9me2, H3K4me3 (to be validated)	26,27
54665	RSBN1 (KDM9)	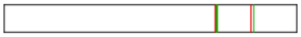	Unknown	

55806	HR	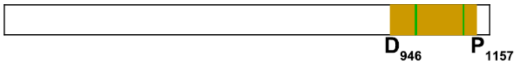	H3K9me1/me2	28
: Catalytic domain; : JmjC domain; :Fe^{2+} binding sites; :2OG binding sites; : JmjN domain				

[*] some JmjC KDMs also have N-methyl arginine residue demethylase activity, though this needs to be validated in cells[29].

[**] Lists of (potential) susbtrates are not exhaustive / some potential substrates need to be validated. The activity of some JmjC KDMs may be regulated by histone modifications distinct from those undergoing demethylation[22].

1. Lu T, Jackson MW, Wang B, et al. Regulation of NF-kappaB by NSD1/FBXL11-dependent reversible lysine methylation of p65. *Proc Natl Acad Sci USA.* 2010;107(1):46-51.
2. Tsukada Y-i, Fang J, Erdjument-Bromage H, et al. Histone demethylation by a family of JmjC domain-containing proteins. *Nature.* 2006;439(7078):811-816.
3. Frescas D, Guardavaccaro D, Bassermann F, Koyama-Nasu R, Pagano M. JHDM1B/FBXL10 is a nucleolar protein that represses transcription of ribosomal RNA genes. *Nature.* 2007;450(7167):309-313.
4. Yamane K, Toumazou C, Tsukada Y-i, et al. JHDM2A, a JmjC-containing H3K9 demethylase, facilitates transcription activation by androgen receptor. *Cell.* 2006;125(3):483-495.
5. Whetstine JR, Nottke A, Lan F, et al. Reversal of histone lysine trimethylation by the JMJD2 family of histone demethylases. *Cell.* 2006;125(3):467-481.
6. Bavetsias V, Lanigan RM, Ruda GF, et al. 8-Substituted Pyrido[3,4-d]pyrimidin-4(3H)-one Derivatives As Potent, Cell Permeable, KDM4 (JMJD2) and KDM5 (JARID1) Histone Lysine Demethylase Inhibitors. *J Med Chem.* 2016;59(4):1388-1409.
7. Hillringhaus L, Yue WW, Rose NR, et al. Structural and evolutionary basis for the dual substrate selectivity of human KDM4 histone demethylase family. *J Biol Chem.* 2011;286(48):41616-41625.
8. Tu S, Teng Y-C, Yuan C, et al. The ARID domain of the H3K4 demethylase RBP2 binds to a DNA CCGCCC motif. *Nat Struct Mol Biol.* 2008;15(4):419-421.
9. Zhang Y, Yang H, Guo X, et al. The PHD1 finger of KDM5B recognizes unmodified H3K4 during the demethylation of histone H3K4me2/3 by KDM5B. *Protein Cell.* 2014;5(11):837-850.
10. Iwase S, Lan F, Bayliss P, et al. The X-linked mental retardation gene SMCX/JARID1C defines a family of histone H3 lysine 4 demethylases. *Cell.* 2007;128(6):1077-1088.
11. Christensen J, Agger K, Cloos PAC, et al. RBP2 belongs to a family of demethylases, specific for tri-and dimethylated lysine 4 on histone 3. *Cell.* 2007;128(6):1063-1076.
12. Tahiliani M, Mei P, Fang R, et al. The histone H3K4 demethylase SMCX links REST target genes to X-linked mental retardation. *Nature.* 2007;447(7144):601-605.
13. Lee MG, Norman J, Shilatifard A, Shiekhattar R. Physical and functional association of a trimethyl H3K4 demethylase and Ring6a/MBLR, a polycomb-like protein. *Cell.* 2007;128(5):877-887.
14. Agger K, Cloos PAC, Christensen J, et al. UTX and JMJD3 are histone H3K27 demethylases involved in HOX gene regulation and development. *Nature.* 2007;449(7163):731-734.
15. Lan F, Bayliss PE, Rinn JL, et al. A histone H3 lysine 27 demethylase regulates animal posterior development. *Nature.* 2007;449(7163):689-694.
16. Lee MG, Villa R, Trojer P, et al. Demethylation of H3K27 regulates polycomb recruitment and H2A ubiquitination. *Science (New York, NY).* 2007;318(5849):447-450.
17. Hong S, Cho Y-W, Yu L-R, Yu H, Veenstra TD, Ge K. Identification of JmjC domain-containing UTX and JMJD3 as histone H3 lysine 27 demethylases. *Proc Natl Acad Sci USA.* 2007;104(47):18439-18444.
18. De Santa F, Totaro MG, Prosperini E, Notarbartolo S, Testa G, Natoli G. The histone H3 lysine-27 demethylase Jmjd3 links inflammation to inhibition of polycomb-mediated gene silencing. *Cell.* 2007;130(6):1083-1094.
19. Walport LJ, Hopkinson RJ, Vollmar M, et al. Human UTY(KDM6C) is a male-specific Nϵ-methyl lysyl demethylase. *J Biol Chem.* 2014;289(26):18302-18313.

20. Tsukada Y-i, Ishitani T, Nakayama KI. KDM7 is a dual demethylase for histone H3 Lys 9 and Lys 27 and functions in brain development. *Genes Dev.* 2010;24(5):432-437.
21. Qi HH, Sarkissian M, Hu G-Q, et al. Histone H4K20/H3K9 demethylase PHF8 regulates zebrafish brain and craniofacial development. *Nature.* 2010;466(7305):503-507.
22. Horton JR, Upadhyay AK, Qi HH, Zhang X, Shi Y, Cheng X. Enzymatic and structural insights for substrate specificity of a family of jumonji histone lysine demethylases. *Nat Struct Mol Biol.* 2010;17(1):38-43.
23. Loenarz C, Ge W, Coleman ML, et al. PHF8, a gene associated with cleft lip/palate and mental retardation, encodes for an Nepsilon-dimethyl lysine demethylase. *Hum Mol Genet.* 2010;19(2):217-222.
24. Liu W, Tanasa B, Tyurina OV, et al. PHF8 mediates histone H4 lysine 20 demethylation events involved in cell cycle progression. *Nature.* 2010;466(7305):508-512.
25. Zhu Z, Wang Y, Li X, et al. PHF8 is a histone H3K9me2 demethylase regulating rRNA synthesis. *Cell Res.* 2010;20(7):794-801.
26. Wen H, Li J, Song T, et al. Recognition of histone H3K4 trimethylation by the plant homeodomain of PHF2 modulates histone demethylation. *J Biol Chem.* 2010;285(13):9322-9326.
27. Baba A, Ohtake F, Okuno Y, et al. PKA-dependent regulation of the histone lysine demethylase complex PHF2-ARID5B. *Nat Cell Biol.* 2011;13(6):668-675.
28. Liu L, Kim H, Casta A, Kobayashi Y, Shapiro LS, Christiano AM. Hairless is a histone H3K9 demethylase. *FASEB J.* 2014;28(4):1534-1542.
29. Walport LJ, Hopkinson RJ, Chowdhury R, et al. Arginine demethylation is catalysed by a subset of JmjC histone lysine demethylases. *Nat Commun.* 2016;7:11974.
