# Supplementary material for: Exploring links between 2‐oxoglutarate‐dependent oxygenases and Alzheimer's disease
Source: Alzheimers Dement. 2022 Jul 19;18(12):2637–68. doi: 10.1002/alz.12733 (PMC10083964; doi:10.1002/alz.12733)
Supplement: Supplementary file 4 — SUPPORTING INFORMATION [file ALZ-18-2637-s004.pdf]

Supplementary Table 3. Domain organization and substrates of nucleic acid oxygenases in 2OGDDs.

Gene ID	2OGDD	Domain architecture	Substrates / Proposed Substrates*	Ref.
8846	ALKBH1	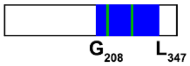	ssDNA, tRNA, dsDNA, mRNA (N-methylated)	1-3
121642	ALKBH2	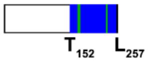	dsDNA (N-methylated)	4,5
221120	ALKBH3	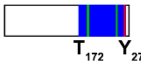	ssDNA, mRNA (N-methylated)	4,6
54890	ALKBH5	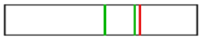	mRNA (N-methylated)	7
91801	ALKBH8	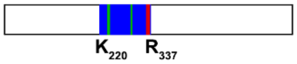	tRNA (hydroxylase)	8,9
79068	FTO	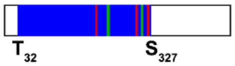	mRNA, tRNA, snRNA (N-methylated)	10,11
129450	TYW5	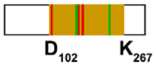	tRNA (hydroxylase)	12
80312	TET1	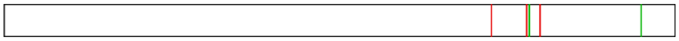	DNA (5-methylcytosine)	13
54790	TET2	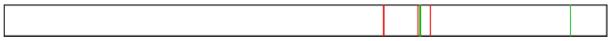	DNA (5-methylcytosine)	14
200424	TET3	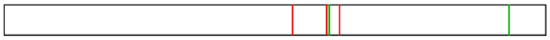	DNA (5-methylcytosine)	15

Supplementary Table 4. Domain organization and substrates of fatty acid and small-molecule oxygenases in 2OGDDs.

Gene ID	2OGDD	Domain architecture	Substrates / Proposed Substrates*	Ref.
8424	BBOX1	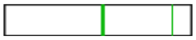	γ-butyrobetaine	16
55217	TMLHE	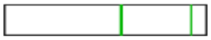	N^{ε}-trimethyllysine	17,18
5262	PHYH (PHYX)	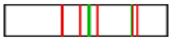	Racemic phytanoyl-CoA, isomers of 3-methylhexadecanoyl-CoA, mono-branched 3-methylacyl-CoA esters (chain length≥C7), straight-chain acyl-CoA esters (chain length ≥ C4)	19,20

Supplementary Table 5. Domain organization of unassigned catalytic function 2OGDDs.

Gene ID	2OGDD	Domain architecture
8424	ALKBH4	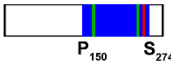
5264	ALKBH6	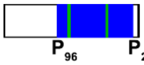
55217	ALKBH7	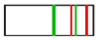
253982	ASPHD1	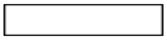
57168	ASPHD2	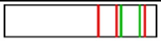
79663	HSPBAP1	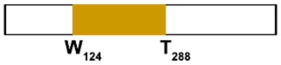
3720	JARID2	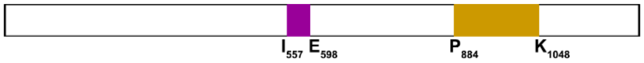
339123	JMJD8	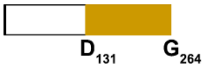
221037	JMJD1C	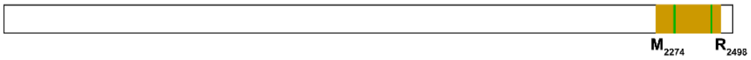

79676	OGFOD2	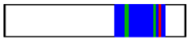
79701	C17orf101 (OGFOD3)	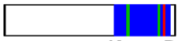
254295	PHYHD1	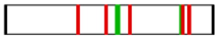
: Catalytic domain : JmjC domain : Fe^{2+} binding sites :2OG binding sites : JmjN domain		

[*] Lists of (potential) substrates are not exhaustive / some potential substrates need to be validated.

1. Müller TA, Meek K, Hausinger RP. Human AlkB homologue 1 (ABH1) exhibits DNA lyase activity at abasic sites. *DNA Repair (Amst).* 2010;9(1):58-65.
2. Westbye MP, Feyzi E, Aas PA, et al. Human AlkB homolog 1 is a mitochondrial protein that demethylates 3-methylcytosine in DNA and RNA. *J Biol Chem.* 2008;283(36):25046-25056.
3. Liu F, Clark W, Luo G, et al. ALKBH1-Mediated tRNA Demethylation Regulates Translation. *Cell.* 2016;167(3).
4. Duncan T, Trewick SC, Koivisto P, Bates PA, Lindahl T, Sedgwick B. Reversal of DNA alkylation damage by two human dioxygenases. *Proc Natl Acad Sci USA.* 2002;99(26):16660-16665.
5. Aas PA, Otterlei M, Falnes PO, et al. Human and bacterial oxidative demethylases repair alkylation damage in both RNA and DNA. *Nature.* 2003;421(6925):859-863.
6. Dominissini D, Nachtergaele S, Moshitch-Moshkovitz S, et al. The dynamic N(1)-methyladenosine methylome in eukaryotic messenger RNA. *Nature.* 2016;530(7591):441-446.
7. Zheng G, Dahl JA, Niu Y, et al. ALKBH5 is a mammalian RNA demethylase that impacts RNA metabolism and mouse fertility. *Mol Cell.* 2013;49(1):18-29.
8. Songe-Møller L, van den Born E, Leihne V, et al. Mammalian ALKBH8 possesses tRNA methyltransferase activity required for the biogenesis of multiple wobble uridine modifications implicated in translational decoding. *Mol Cell Biol.* 2010;30(7):1814-1827.
9. van den Born E, Vågbø CB, Songe-Møller L, et al. ALKBH8-mediated formation of a novel diastereomeric pair of wobble nucleosides in mammalian tRNA. *Nat Commun.* 2011;2:172.
10. Jia G, Fu Y, Zhao X, et al. N6-methyladenosine in nuclear RNA is a major substrate of the obesity-associated FTO. *Nature chemical biology.* 2011;7(12):885-887.
11. Wei J, Liu F, Lu Z, et al. Differential mA, mA, and mA Demethylation Mediated by FTO in the Cell Nucleus and Cytoplasm. *Mol Cell.* 2018;71(6).
12. Noma A, Ishitani R, Kato M, Nagao A, Nureki O, Suzuki T. Expanding role of the jumonji C domain as an RNA hydroxylase. *J Biol Chem.* 2010;285(45):34503-34507.
13. Tahiliani M, Koh KP, Shen Y, et al. Conversion of 5-methylcytosine to 5-hydroxymethylcytosine in mammalian DNA by MLL partner TET1. *Science (New York, NY).* 2009;324(5929):930-935.
14. Langemeijer SMC, Kuiper RP, Berends M, et al. Acquired mutations in TET2 are common in myelodysplastic syndromes. *Nat Genet.* 2009;41(7):838-842.
15. Xu Y, Xu C, Kato A, et al. Tet3 CXXC domain and dioxygenase activity cooperatively regulate key genes for Xenopus eye and neural development. *Cell.* 2012;151(6):1200-1213.
16. Tars K, Rumnieks J, Zeltins A, et al. Crystal structure of human gamma-butyrobetaine hydroxylase. *Biochemical and biophysical research communications.* 2010;398(4):634-639.
17. Vaz FM, Ofman R, Westinga K, Back JW, Wanders RJ. Molecular and Biochemical Characterization of Rat epsilon -N-Trimethyllysine Hydroxylase, the First Enzyme of Carnitine Biosynthesis. *J Biol Chem.* 2001;276(36):33512-33517.
18. Nava C, Lamari F, Héron D, et al. Analysis of the chromosome X exome in patients with autism spectrum disorders identified novel candidate genes, including TMLHE. *Translational psychiatry.* 2012;2:e179.
19. Croes K, Foulon V, Casteels M, Van Veldhoven PP, Mannaerts GP. Phytanoyl-CoA hydroxylase: recognition of 3-methyl-

branched acyl-coAs and requirement for GTP or ATP and Mg(2+) in addition to its known hydroxylation cofactors. *J Lipid Res.* 2000;41(4):629-636.

20. Foulon V, Asselberghs S, Geens W, Mannaerts GP, Casteels M, Van Veldhoven PP. Further studies on the substrate spectrum of phytanoyl-CoA hydroxylase: implications for Refsum disease? *J Lipid Res.* 2003;44(12):2349-2355.
